# Chamelogk: A Chromatographic Chameleonicity Quantifier to Design Orally Bioavailable Beyond-Rule-of-5 Drugs

**DOI:** 10.1021/acs.jmedchem.3c00823

**Published:** 2023-07-25

**Authors:** Diego Garcia Jimenez, Maura Vallaro, Matteo Rossi Sebastiano, Giulia Apprato, Giulia D’Agostini, Paolo Rossetti, Giuseppe Ermondi, Giulia Caron

**Affiliations:** Molecular Biotechnology and Health Sciences Dept., CASSMedChem, University of Torino, via Quarello 15, 10135 Torino, Italy

## Abstract

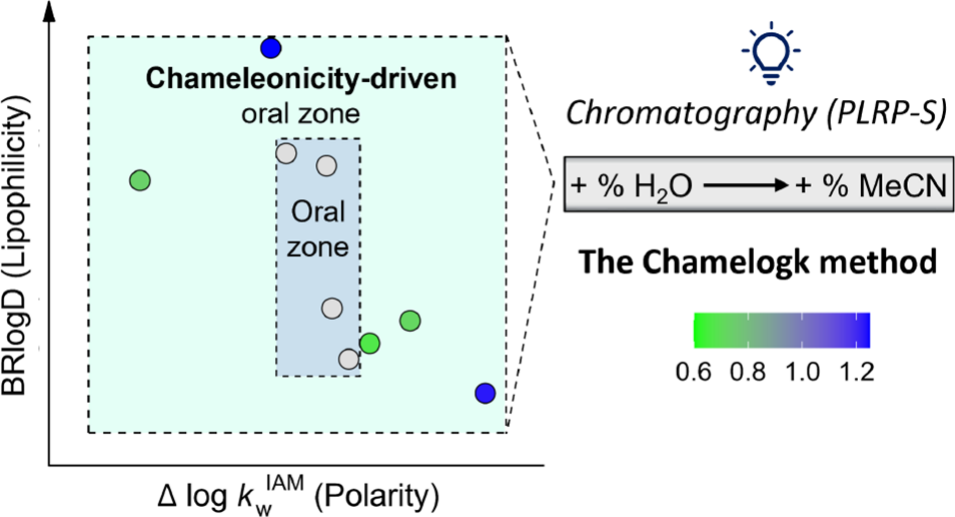

New chemical modalities in drug discovery include molecules belonging to the bRo5 chemical space. Because of their complex and flexible structure, bRo5 compounds often suffer from a poor solubility/permeability profile. Chameleonicity describes the capacity of a molecule to adapt to the environment through conformational changes; the design of molecular chameleons is a medicinal chemistry strategy simultaneously optimizing solubility and permeability. A default method to quantify chameleonicity in early drug discovery is still missing. Here we introduce Chamelogk, an automated, fast, and cheap chromatographic descriptor of chameleonicity. Moreover, we report measurements for 55 Ro5 and bRo5 compounds and validate our method with literature data. Then, selected case studies (macrocycles, nonmacrocyclic compounds, and PROTACs) are used to illustrate the application of Chamelogk in combination with lipophilicity (BRlogD) and polarity (Δ log *k*_w_^IAM^) descriptors. Overall, we show how Chamelogk deserves being included in property-based drug discovery strategies to design oral bioavailable bRo5 compounds.

## Introduction

Drug discovery has been dramatically changing in the last years because of the large increase in the number of drug candidates residing in the chemical space outside of Lipinski’s rule of 5, i.e., the so-called beyond-rule-of-5 (bRo5) chemical space.^1,2^ Two main reasons can explain this trend: (a) the modulation of difficult-to-drug targets is more likely to be achieved with large bRo5 molecules^3^ than small Ro5-compliant compounds, and (b) within the bRo5 chemical space, degraders, also termed proteolysis targeting chimeras (PROTACs), have generated wide interest because of their innovative mode of action and huge potential to treat unmet diseases.^4,5^

It is widely known that promising drug candidates often fail to reach further development because of unsatisfactory ADME properties (Absorption, Distribution, Metabolism, and Excretion) resulting in poor oral bioavailability (e.g., low cell permeability and solubility).^6^ bRo5 molecules have large and flexible structures and thus are prone to suffering from major ADME limitations, being too large for concomitant solubility and cell permeability.^1^ The relationship and interplay between solubility and permeability make their simultaneous optimization a challenge for medicinal chemists in any drug discovery program.^7^ For example, increasing permeability by increasing lipophilicity may decrease solubility and metabolic stability. The chemical space transfer from Ro5 to bRo5 often complicates the measurement, modeling, and prediction of the permeability/solubility pair and related physicochemical descriptors like lipophilicity and polarity.^8^ In practice, obtaining orally available bRo5 molecules is more difficult than in the traditional Ro5 space.^3,9,10^

Matsson and co-workers^11^ hypothesized that by maintaining Ro5-like heavy-atom/carbon ratios, drugs and clinical candidates larger than 700 Da need to undergo conformational changes to adapt their physicochemical properties to the environment. This is commonly referred to as chameleonic behavior. This was somewhat definitively stated by Whitty and co-workers who claimed that a certain degree of chameleonicity is needed for high MW to become oral drugs.^12^ Although Carrupt et al. already introduced this concept in the 1990s to justify the peculiar pharmacokinetic properties of morphine glucuronides,^13^ the rising star of this theory is the macrocycle cyclosporine (CsA). Formally, macrocycles are molecules containing a ring of at least 12 heavy atoms, which display remarkable pharmacodynamic properties due to their capacity to bind to “difficult to drug” binding sites.^14^

However, macrocycles still suffer from DMPK limitations and often require chameleonic properties to be orally available. Thus, the study of CsA as the first example of a chameleonic macrocycle led Alex et al.^15^ to hypothesize that the unexpectedly high permeability of CsA is due to a conformational change from an extended conformation in water (where the backbone amides mostly form intermolecular hydrogen bonds (HBs) with the solvent) to a more folded conformation in the membrane interior (where intramolecular hydrogen bonds (IMHBs) are formed). In fact, some studies highlight the need to display congruent conformations (equivalent conformations in polar and nonpolar media) to lower the price that closed conformations should pay when passing from nonpolar to polar environments.^16^ Additional studies were published to further inspect macrocycle chameleonicity.^16−21^ For instance, Rossi Sebastiano and co-workers^22^ used a data set of crystallographic drug structures to highlight that dynamic IMHBs (dIMHBs) and hydrophobic collapse are two structural chameleonicity drivers.^23^ Besides macrocycles, chameleonicity may also affect other bRo5 classes such as nonmacrocyclic compounds and PROTACs (often referred to as degraders), defined as heterobifunctional molecules composed of three parts: a warhead targeting a protein of interest (POI), an E3 ligand recruiting an E3 ligase enzyme, and a linker connecting both regions.^4^ Degraders have become popular in drug discovery because of their innovative mechanism of action able to modulate the “undruggable” but are even further away from the oral Ro5 space than most macrocycles,^24^ with the consequent DMPK issues. Thus, the applicability of chameleonicity to PROTACs has gained relevance, as proven by Kihlberg’s group.^25,26^ Not long ago, our team proved that saquinavir, an orally available nonmacrocyclic bRo5 drug, can also exhibit this behavior.^27^ Very recently, new chameleonicity-dependent models to explain cyclic decapeptide cellular-passive permeability theories have been proposed.^10^ Moreover, a few very interesting papers have been reported about cyclic peptide (CP) structure–permeability relationships.^28−31^ However, the complex and peculiar structural features of CPs (canonical amino acid and noncanonical element composition, secondary structure motifs, IMHB backbone driven, N-methylation, etc.) suggested not including them in this paper but rather dedicating a specific study later.

Overall, the recent literature suggests that to expand the pool of medicinal chemistry strategies aimed at simultaneously optimizing solubility and permeability, there is a need for experimental methods capable of quantifying chameleonicity. To date, chameleonicity has been tentatively quantified in different ways. A first tool is X-ray crystallography that involves the analysis of the crystallized conformers reported in online databases like the Protein Data Bank (PDB)^32^ or the Cambridge Structural Database (CSD).^32^ In short, the conformers are superimposed and molecular properties are calculated. The most common molecular descriptor is 3D-PSA, the calculated polar surface area of a 3D conformer.^22^ Once this is done, the property window obtained by the difference between the maximum and minimum 3D-PSA is used as a numerical value to express chameleonic behavior. This is usually verified by comparison with known standards and by the analysis of intramolecular interactions in representative conformations.^22,33^ Ideally, conformers should have been crystallized from solvents with different polarity, but this is not often verified. However, distinct conformers can be extracted from protein-bound co-crystals, in which the pocket’s nature represents the environmental variable. This approach suffers from several limitations and weaknesses: the small number of available crystallized structures, the crystal packing effects (it is not guaranteed that conformations in the solid state are also present in solution), and the underestimation of chameleonicity since it is never certain that the conformers with extreme properties were crystallized.

A second approach to evaluate chameleonicity is to use NMR to assess conformational ensembles in solutions. Then, similar to that described above for crystallography, conformers are characterized by molecular properties. In this case, the solvent can be *ad hoc* chosen to mimic different polarities accounting for the *in vivo* situations. Using this technique, two macrocycles, telithromycin^34^ and roxithromycin,^33,34^ were identified as true chameleons, whereas rifampicin^34,35^ was revealed to be a weak chameleon. Indeed, NMR showed that the orientation of the side chains of telithromycin and roxithromycin varied between nonpolar and polar environments, confirming the crucial effect of macrocyclic side chains on chameleonicity.^22^ Recently, the same method has also been applied to PROTACs^25^ and nonmacrocyclic compounds (antivirals).^36^ This approach has the advantage of focusing on true solution conformers, but it mainly remains a semiquantitative case-per-case investigation method. Moreover, it is time-consuming, and it requires specialized expertise, making it unsuitable for HT drug discovery applications. Finally, bRo5 compounds often have solubility issues in media other than DMSO.

The third experimental and published tool to quantify chameleonicity is ChamelogD, which may be considered an HT method based on chromatographic measurements.^37^ ChamelogD is defined as the difference between ElogD^38^ and BRlogD,^39^ two chromatographic indexes obtained in different environments. The greater the difference between the two indexes is, the more chameleonic a compound is. Its main limitation concerns the inability to extract the populated conformers in each environment since it just provides a numerical value that represents a behavioral change of the conformational ensemble.

Finally, we need to mention that some computational efforts were made to try to quantify chameleonicity;^27^ however, a full computational reproducibility of experimental data is not yet feasible.

According to the above discussion, a standard method for a rapid and simple quantification of chameleonicity in early drug discovery is still missing. In this paper, we introduce Chamelogk, an experimental chromatographic descriptor of chameleonicity, and report Chamelogk values for a data set of 55 Ro5 and bRo5 compounds. Then, we define a threshold for distinguishing chameleonic from nonchameleonic molecules and use literature results to validate our method. In the last part of the paper, we suggest, through selected case studies, how to apply Chamelogk in drug design. Specifically, we highlight the applicability of chameleonicity on the basis of the lipophilicity/polarity profile of the investigated compounds. Overall, we show how Chamelogk is a powerful descriptor of chameleonicity and deserves to be included in property-based drug discovery strategies for bRo5 compounds.

## Results and Discussion

### Chamelogk: The Method and Its Design

**Figure 1:**
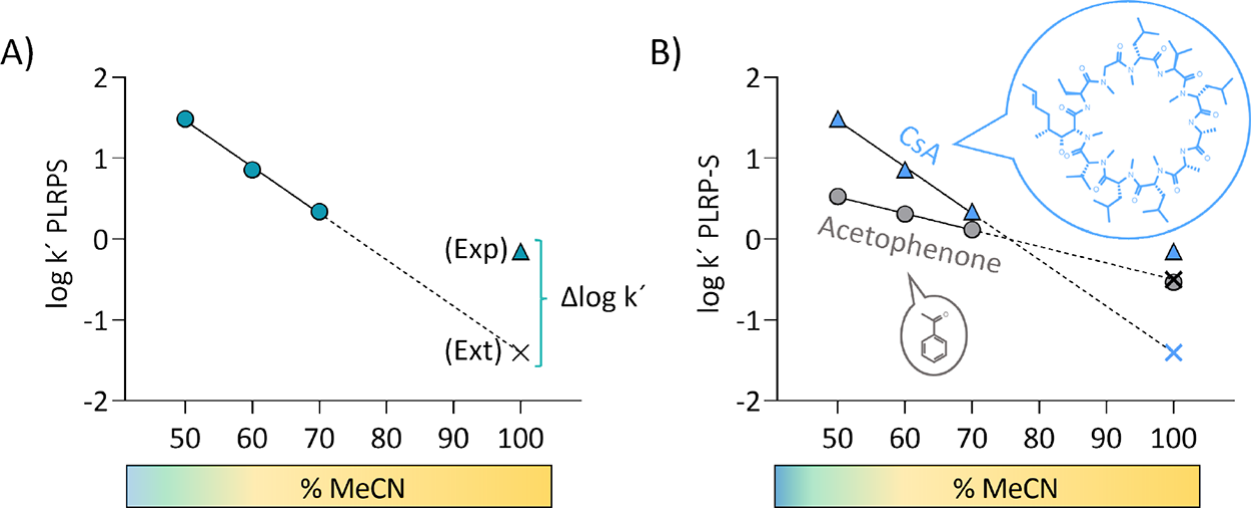
(A) Graphical scheme of Chamelogk. It is calculated as the difference between the experimental (Exp, colored triangle) and the extrapolated value (Ext, presented as a black cross) at 100% MeCN. (B) Chamelogk plot of cyclosporine (CsA, neutral bRo5, blue triangles) and acetophenone (neutral Ro5 compound, gray circles). Exp. log k’PLRP-S and Ext. log k’100 PLRP-S values are presented as colored symbols and colored crosses, respectively.

Considering the need for a chameleonicity quantifier to be used in very early drug discovery, we set out to develop a method focusing on reverse-phase liquid chromatography (RP-HPLC) that would be fast enough for medium- to high-throughput applications, insensitive to impurities, and amenable to automation. In particular, we decided to focus on an RP-HPLC system with a unique stationary phase. Ideally, we intended to create a dynamic environment that mimicked the journey of a molecule through the cell membrane. For this purpose, we used water (dielectric constant ε ∼80) and acetonitrile (MeCN, dielectric constant ε ∼37.5) in different proportions as mobile phases and searched for a stationary phase that could provide a nonpolar environment similar to the interior of the cell. In particular, we focused on a polystyrene/divinylbenzene polymeric column (PLRP-S) already used^40^ to assess the well-known log P in toluene/water (log P_tol_) at 80% MeCN (log k’80 PLRP-S).^40^ In practice, with a less polar mobile phase (100% MeCN), we would guarantee an almost fully nonpolar environment that is expected to simulate the largely nonpolar interior of the cell membrane. In previous studies,^27,41^ we monitored the variation of the logarithm of the capacity factor (log k’ PLRP-S) with the mobile phase composition, and we verified that few bRo5 molecules (i.e., CsA, saquinavir,^27^ MZ1,^41^ and PROTAC-1^42^) showed a different behavior than Ro5 compliant compounds (e.g., pomalidomide^27^). The latter respected the reverse-phase nature of the PLRP-S chromatographic system because the retention time (and thus log k’ PLRP-S) of lipophilic molecules decreases when the amount of MeCN in the mobile phase increases (Figure S1). However, for some bRo5 candidates, we observed a deviation from the linear trend at high MeCN% values (>70%), maximized at 100% MeCN (Figure S1). This experimental evidence suggested defining a simple descriptor that quantifies the different behavior exhibited by the investigated structures in the PLRP-S system. To do that, once a compound is selected, the first step involves the experimental measurement of log k’PLRP-S values at 50, 60, and 70% of MeCN. A linear fitting between log k’PLRP-S and the % MeCN can be obtained with an expected high *R*^2^ (*R*^2^ ≥ 0.90, Figure 1A). This linear regression is used to obtain an extrapolated log k’PLRP-S value at 100% MeCN (named Ext. log k’PLRP-S). Notably, Chamelogk should be reported with the *R*^2^ value of the linear trend (50–70% MeCN) to check the reliability of the Chamelogk value. Finally, we experimentally measure the log k’PLRP-S value at 100% MeCN (named Exp. Log k’PLRP-S). We defined Chamelogk as the capacity factor difference (Δ log k’) between the experimental log k measured with 100% MeCN (Exp. Log k’100) and the extrapolated correspondent value (Ext. log k’100, obtained from log k’ PLRP-S values at 50, 60, and 70% of MeCN), as reflected by eq 1 and Figure 1A.
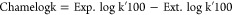
1

According to the Chamelogk definition, nonchameleonic compounds are expected to show low Chamelogk values, whereas molecular chameleons are expected to show larger values. Our interpretation for this is that in nonpolar environments, chameleons adapt their conformations by reducing the exposed polar surface area and thus increasing their log k’ PLRP-S value. Figure 1B shows the different behavior of acetophenone (Ro5) and cyclosporine (bRo5 and macrocyclic) (Table S1) for which Chamelogk values of −0.04 and 1.25 were respectively obtained. As shown by the graph (Figure 1B), both compounds suffer a constant retention time reduction when increasing the MeCN contribution from 50 to 70% described by a linear trend (*R*^2^ > 0.90). However, at 100%, acetophenone maintains the linear trend, whereas cyclosporine is more retained to the stationary phase of the column. This behavior is expected to be a result of a property change probably due to a conformational change in nonpolar environments.

### Chamelogk Data Collection

In this study, the Chamelogk experiment was performed for a data set of 55 commercially available neutral molecules (expected to be at least 50% neutral) and classified according to their Ro5 or bRo5 nature (class) and substructure (subclass) (Table 1). The Ro5 class was further divided into classical Ro5 compounds and PROTAC building blocks (i.e., E3 ligands, warheads, and linkers), whereas bRo5 were divided into three subclasses: macrocycles (cyclic structure with ≥12 heavy atoms), nonmacrocyclic bRo5 compounds, and PROTACs. Notably, some complex PROTAC building blocks also belong to the bRo5 space (i.e., PEG_4_-PH-NH_2_-pomalidomide). Chamelogk and *R*^2^ values are in Table 1. Overall, Chamelogk ranges from −0.22 (hydrochlorothiazide) to 1.36 (gefitinib-based PROTAC 3) with a median value of 0.45.

**Table 1:** Chamelogk Measurements for Neutral Ro5 and bRo5 Compounds (*N* = 55)^a^

compound	class	subclass	Chamelogk	*R*^2^	MW	TPSA	PHI
3-bromoquinoline	Ro5	classic Ro5	0.13	1.00	208	13	2
acetone	Ro5	classic Ro5	–0.13	0.96	58	17	1
acetophenone	Ro5	classic Ro5	–0.04	1.00	120	17	2
bifonazole	Ro5	classic Ro5	0.45	1.00	310	18	4
clotrimazole	Ro5	classic Ro5	0.71	0.99	345	18	4
diazepam	Ro5	classic Ro5	0.30	1.00	285	33	3
diethylstilbestrol	Ro5	classic Ro5	0.44	1.00	268	40	5
hydrochlorothiazide	Ro5	classic Ro5	–0.22	1.00	298	135	3
hydrocortisone	Ro5	classic Ro5	0.10	0.91	363	95	4
naphthalene	Ro5	classic Ro5	0.02	1.00	128	0	1
phenol	Ro5	classic Ro5	0.16	1.00	94	20	1
toluene	Ro5	classic Ro5	0.06	0.99	92	0	1
4-F-thalidomide	Ro5	E3 ligand	0.23	1.00	276	85	3
4-hydroxy thalidomide	Ro5	E3 ligand	–0.16	0.84	274	105	3
*cis*-OH-VH298 (S,S,S)	Ro5	E3 ligand	0.45	1.00	540	184	8
*cis*-phenol-VH032 (S,S,S)	Ro5	E3 ligand	0.65	0.99	489	160	8
OH-VH298 (S,R,S)	Ro5	E3 ligand	0.48	1.00	540	184	8
phenol-VH032 (S,R,S)	Ro5	E3 ligand	0.37	1.00	489	160	8
pomalidomide	Ro5	E3 ligand	0.00	1.00	273	111	3
BI-0115	Ro5	warhead	0.19	0.99	288	51	4
BI-1580	Ro5	warhead	0.10	0.99	253	51	3
CPI203	Ro5	warhead	0.54	0.98	400	114	5
HJB97	Ro5	warhead	0.15	0.99	501	136	6
MS-417	Ro5	warhead	0.29	0.97	415	98	5
OTX-015	Ro5	warhead	0.64	0.97	492	121	6
cyclosporine	bRo5	macrocycle	1.25	1.00	1203	279	34
everolimus	bRo5	macrocycle	0.45	1.00	958	205	23
pimecrolimus	bRo5	macrocycle	0.43	0.99	811	158	17
sirolimus	bRo5	macrocycle	0.25	1.00	914	195	21
temsirolimus	bRo5	macrocycle	0.23	1.00	1030	242	24
atazanavir	bRo5	nonmacrocycle	0.33	1.00	705	171	15
nelfinavir	bRo5	nonmacrocycle	0.74	1.00	568	127	11
paclitaxel	bRo5	nonmacrocycle	0.15	1.00	854	221	12
ritonavir	bRo5	nonmacrocycle	0.67	1.00	721	202	16
saquinavir	bRo5	nonmacrocycle	1.23	1.00	671	167	12
telaprevir	bRo5	nonmacrocycle	0.31	1.00	680	180	12
PEG_4_-PH-NH_2_-pomalidomide	bRo5	E3 ligand	0.12	1.00	541	160	11
ARV-825	bRo5	PROTAC	0.72	0.98	924	235	15
BI-0319	bRo5	PROTAC	0.99	1.00	1061	270	20
BI-3663	bRo5	PROTAC	0.50	1.00	918	244	16
BI-4206	bRo5	PROTAC	0.70	0.99	1061	270	20
BRD4 degrader AT1	bRo5	PROTAC	1.26	0.99	973	266	17
*cis*MZ1	bRo5	PROTAC	1.27	0.98	1003	268	18
CRBN-6-5-5-VHL	bRo5	PROTAC	1.05	1.00	972	256	20
dBET1	bRo5	PROTAC	0.80	0.99	785	224	11
dBET57	bRo5	PROTAC	0.68	1.00	699	198	9
dBET6	bRo5	PROTAC	0.86	0.99	841	224	14
gefitinib-based PROTAC 3	bRo5	PROTAC	1.36	0.98	935	215	18
MZ1	bRo5	PROTAC	1.15	0.99	1003	268	18
MZP-54	bRo5	PROTAC	1.18	1.00	1037	229	20
PROTAC BET degrader-10	bRo5	PROTAC	0.83	0.99	783	215	11
PROTAC FAK degrader-1	bRo5	PROTAC	0.79	0.99	996	254	18
PROTAC Mcl degrader-1	bRo5	PROTAC	0.93	0.99	910	220	15
PROTAC-1	bRo5	PROTAC	1.07	0.99	1034	265	19
ZXH-3-26	bRo5	PROTAC	0.65	0.99	785	224	11

a Entries were ordered sequentially by class, subclass, and Chamelogk. The classification into Ro5 and bRo5 was based on Lipinski’s guidelines.^43^ The Ro5 class was defined to have just one violation of the following: MW < 500 Da and no more than 5 and 10 HBD and HBA, respectively. Nelfinavir was manually classified as a bRo5 drug despite being formally Ro5 compliant. This was due to the violation of Veber’s guidelines and similarity to the bRo5 antiviral series. MW, TPSA (topological polar surface area), and PHI (Kier’s flexibility index) are reported as descriptors of size, polarity, and flexibility, respectively; a complete set of 2D descriptors is provided in Table S2.

**Figure 2:**
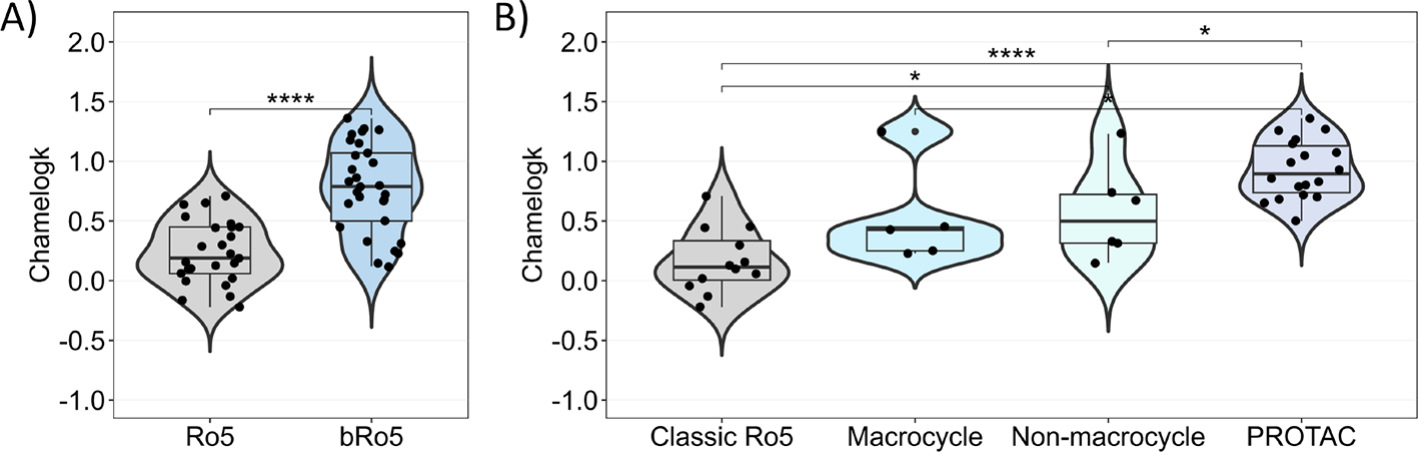
(A) Chamelogk distribution of neutral Ro5 (*n* = 25) and bRo5 compounds (*n* = 30). (B) Chamelogk distribution of bRo5 subclasses (*n* = 29): macrocycle (*n* = 5), nonmacrocycle (*n* = 6), and PROTAC (*n* = 18). For comparative purposes, only classical Ro5 compounds were displayed (*n* = 12) (E3 ligands and warheads were removed). Statistical significance is presented as *p* values from Wilcoxon’s test: 0–0.0001 (****), 0001–0.001 (***), 0.001–0.01 (**), 0.01–0.05 (*), 0.05–1 (ns).

Although we are aware that the data set does not represent any drug chemical space, we performed some statistical analyses to at least gain insights on the main data set trends. Figure 2A shows that Ro5 compliant molecules display significantly lower Chamelogk values than bRo5 derivatives (median values 0.19 and 0.77, respectively). Figure 2B allows one to individually compare the three main subclasses of bRo5 compounds (PROTACs, macrocycles, and nonmacrocycles) with classical Ro5 ones (median value 0.12). PROTACs and nonmacrocycles showed significantly higher Chamelogk values (median values 0.90 and 0.5, respectively), whereas macrocycles seemed to exhibit poorer chameleonic properties (median value 0.43).

**Figure 3:**
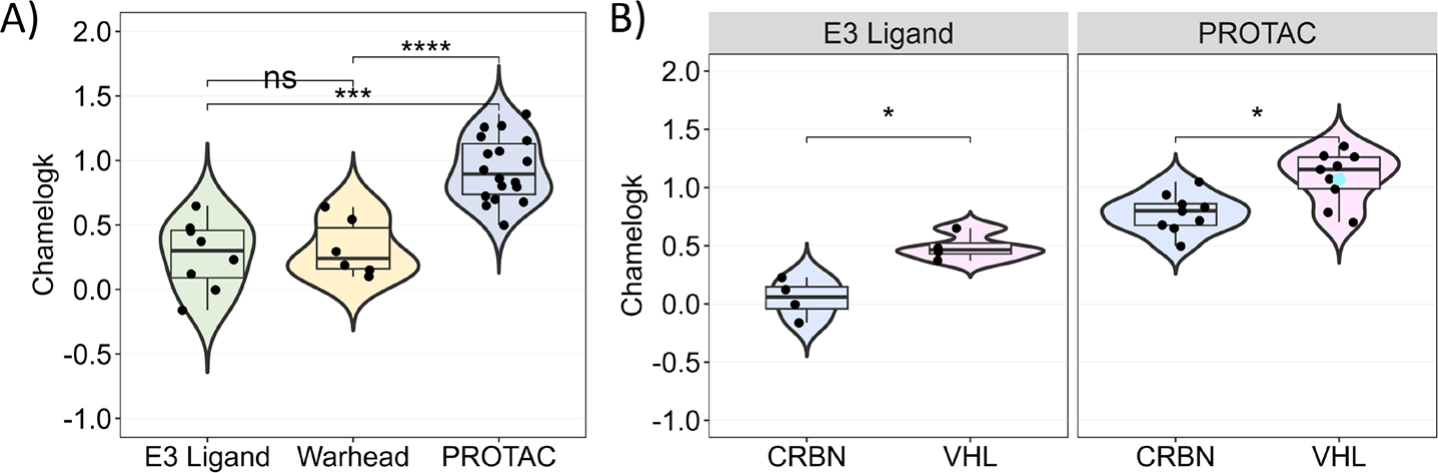
(A) Chamelogk distribution of E3 ligands (*n* = 8), warheads (*n* = 6), and PROTACs (*n* = 18). (B) Panel 1: Chamelogk distribution of E3 ligands (*n* = 8) (CRBN, *n* = 4; VHL, *n* = 4). Panel 2: PROTACs (*n* = 18) (CRBN-based, *n* = 9; VHL-based, *n* = 9). PROTAC-1 is presented as a light-blue dot. The E3 ligand-binder subclassification for the E3 ligand and PROTAC subsets can be found in Table S4. Statistical significance is presented as *p* values: 0–0.0001 (****), 0001–0.001 (***), 0.001–0.01 (**), 0.01–0.05 (*), 0.05–1 (ns) (Wilcoxon’s test).

The next step of our study was to perform a deeper analysis on the most populated bRo5 subclass in our data set, i.e., PROTACs. First, we focused on the constitutive building blocks, namely, E3 ligands and warheads (Figure 3A): most of them belong to the Ro5 space, and indeed, no significant difference was found in the median values (0.30 and 0.24, respectively). According to the PROTAC definition (see Introduction), whereas the warhead’s contribution is somehow heterogeneous, the E3 ligands of our data set are either (a) pomalidomide or thalidomide derivatives (cereblon (CRBN) binders) or (b) VHL-ligand derivatives (Von Hippel–Lindau (VHL) binders). CRBN binders are generally smaller, more rigid, and slightly less polar (Table S3 compares pomalidomide and VH-032). Because any descriptor comparison between pomalidomide and VHL-032 is biased by the different ionization^44^ profiles, we focused on Chamelogk for neutral VHL derivatives and verified that VHL binders are statistically more chameleonic than the CRBN ones (median values 0.47 and 0.06, respectively, Figure 3B). This trend is also verified for the corresponding PROTACs (median values 1.16 and 0.8, respectively).

### Chamelogk Interpretation and Validation with Literature Data

**Figure 4:**
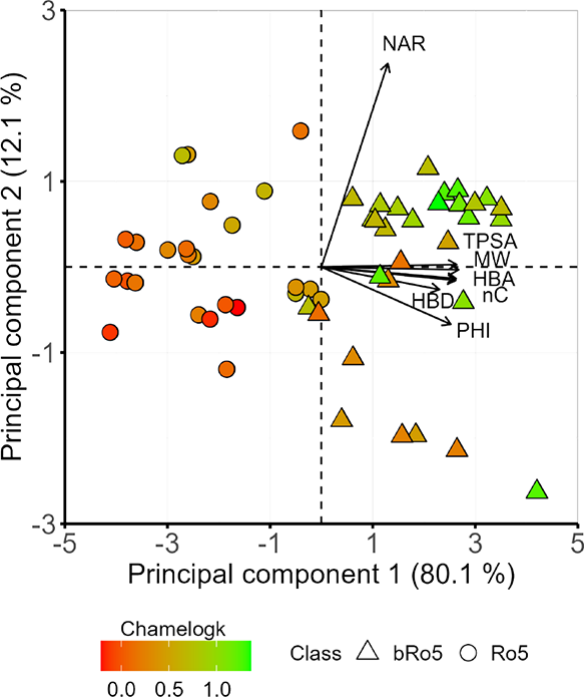
Chameleonicity distribution (red-green color scale) according to a PCA for the 2D molecular descriptors of the neutral set (*n* = 55). The contributions of individual descriptors to the PCAs are indicated by the length of the arrows. Ro5 compounds are presented as circles, whereas bRo5 subclasses are presented as triangles. Vertical and horizontal jitter (0.13 PC1 and PC2 units) was introduced to avoid point overlap.

Chamelogk is intuitively associated to the concept of chameleonicity (see above), but this should be confirmed on the basis of a molecular property rationale. Overall, we assume that chameleonicity is expected to increase with molecular complexity and thus needs to be evaluated by structural descriptors. Some of us^24^ reported a set of simple 2D molecular descriptors useful to depict structural differences for the bRo5 space (Table S2): MW and the number of carbons (nC) report size, TPSA (topological polar surface area), HBA and HBD polarity, PHI (Kier’s flexibility index) flexibility, and the number of aromatic ring (NAR) lipophilicity.^24^ Notably, nC can also be related to the nonpolar part of the molecule as a hydrophobicity index. Thus, we performed a PCA with the seven molecular descriptors.^24^ The scores/loadings plots are presented as a biplot in Figure 4.

Principal components 1 and 2 explain approximately 90% of the descriptor variability with PC3 explaining 5%. PC1 is mainly driven by MW, TPSA, nC, HBA, and PHI, whereas PC2 and PC3 are guided by NAR and HBD, respectively. Thus, size, polarity and flexibility seem to guarantee an increase in Chamelogk. Moreover, red and orange dots suggest that small and nonpolar molecules are decisively nonchameleonic, in agreement with the Ro5 classification (circles in Figure 4). Overall, chameleonic drugs show greater size, polarity, and flexibility, necessary for structural chameleonicity. This behavior proves that Chamelogk is a feature almost exclusive to the bRo5 chemical subspace. As expected, no patterns are observed within individual bRo5 subclasses. As introduced earlier,^27^ factors underlying Chamelogk differences among the bRo5 space require a complete *in silico* conformational characterization, which is beyond the scope of this paper.

The next step of the study consisted of validating our findings with experimental chameleonicity data reported in the literature. In particular, we focused on a series of bRo5 molecules for which X-ray, NMR, or ChamelogD data (see Introduction) are available. However, we are aware that each experimental technique has limitations (i.e., solvent solubility, charge handling, crystallization capacity, etc.) and sometimes impedes complete replication with other techniques. Consequently, we can only evaluate whether Chamelogk can identify highly chameleonic compounds previously reported by other groups and distinguish weak from strong chameleons. Table 2 provides a list of suspected and/or confirmed chameleons reported in the literature and the methods applied to monitor their chameleonic behavior.

**Table 2:** Chameleonicity Assessment of Neutral bRo5 Compounds Classified by bRo5 Subtypes and Ordered in Increasing Value of Chamelogk

**Compound**	**Subclass**	**ChameLogD**(37)	**Chamelogk**	**X-Ray**		**NMR**(20,25)
				**Δ 3D-PSA**(22)	**Crystal analysis**(16,17,19−22)	
**Temsirolimus**	Macrocycle	2	0.23	ND		
**Sirolimus**	Macrocycle	1.4	0.25	ND		
**Everolimus**	Macrocycle	1.7	0.45	ND		
**Cyclosporine**	Macrocycle	2.3	1.25	79	Chameleon	Chameleon
**Paclitaxel**	Nonmacrocycle	0.3	0.15	23		
**Telaprevir**	Nonmacrocycle	0.9	0.31	32		
**Atazanavir**	Nonmacrocycle	1.6	0.33	34		
**Ritonavir**	Nonmacrocycle	1.6	0.67	53		
**Nelfinavir**	Nonmacrocycle	1.4	0.74	ND		
**Saquinavir**	Nonmacrocycle	2.3	1.23	21		
**PROTAC-1**	PROTAC	ND	1.07	ND		Chameleon

**Figure 5:**
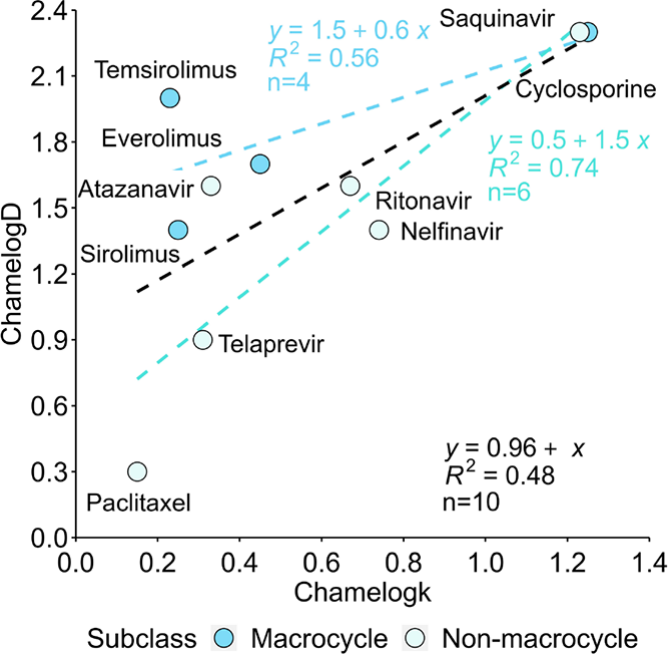
ChamelogD vs Chamelogk (*n* = 10). Dashed lines represent the linear regression for the neutral bRo5 compounds (black), macrocycles (blue) and nonmacrocyclic bRo5 compounds (light blue).

First, we compared Chamelogk with ChamelogD previously reported by our group (Table 2).^37^ The two methods show a fair linear correlation (Figure 5) (*R*^2^ = 0.48) when macrocyclic and nonmacrocyclic bRo5 compounds are considered together. If nonmacrocyclic compounds are considered separately, the relationship strongly improves (*R*^2^ = 0.74, *n* = 6). Moreover, both agree on the high chameleonicity of saquinavir and cyclosporine. Saquinavir is a nonmacrocyclic bRo5 compound recently studied by our group,^27^ proven to be chameleonic by formation of IMHBs, and cyclosporine is a widely known chameleon, confirmed both by X-ray^16,17,19−22^ and NMR,^20^ as discussed in the Introduction.

Both Chamelogk and ChamelogD are obtained by chromatographic techniques, but Chamelogk is in our opinion superior to ChamelogD because it explores different environments within the same stationary phase. Moreover, ChamelogD requires two different chromatographic systems (BRlogD and ElogD), and one of them is an extrapolated value (ElogD). In practice, Chamelogk is faster and more reproducible than ChamelogD.

Next, we focused on the crystallographic approach to describe chameleonicity. As a general trend, it is accepted that a low structural superposition (high root mean square deviation (RMSD)) and a large molecular property window (radius of gyration or RGyr, 3D-PSA, etc.) among the crystallized conformers are indicators of chameleonicity.^22,33^ To validate Chamelogk, we first focused on the polarity difference (Δ 3D-PSA) as calculated among crystallized conformers within the subset reported by Rossi Sebastiano and coworkers (macrocyclic and nonmacrocyclic).^22^ As previously introduced,^22^ a high Δ 3D-PSA suggests a high chameleonicity. However, we could only find a poor correlation between Chamelogk and Δ 3D-PSA in the data set (*R*^2^ = 0.23, Figure S2). For instance, there is agreement on cyclosporine^22^ but disagreement on saquinavir (proven chameleon by ChamelogD,^37^Figure 5). In our opinion, some disagreement can reside in the fact that the identification of a compound as chameleon by X-ray is an imperious proof (e.g., cyclosporine), but the absence of different conformations cannot be taken as absolute proof of no chameleonicity. In short, one can never be sure that all possible conformations have been crystallized, thus preventing the identification of negative controls.

Finally, NMR techniques combined with the NMR analysis of molecular flexibility in solution (NAMFIS) algorithm reveal the most probable conformations in both polar and nonpolar solutions.^34^ Thus, Chamelogk agrees with NMR on cyclosporine^20^ (as expected) and PROTAC-1,^25^ a neutral and cell-permeable degrader. However, the paucity of NMR studies limits the comparative analysis with this methodology.

Overall, except for the differences discussed, Chamelogk agrees with most literature data quantifying chameleonicity of neutral compounds.

### Chamelogk Threshold for the Identification of a Chameleon

An obvious and sound question is what value of Chamelogk can distinguish molecular chameleons from other molecules. In fact, all flexible compounds can in principle adopt different conformers in different environments, but not all of them will significantly impact their properties.^27^ The identification of a Chamelogk threshold is not trivial, mainly because its value is affected by the overall structural complexity of compounds, as shown by Figures 3. and 4 Of note, such differences can be inter-class (Ro5 vs bRo5) and arising from intra-subclass structural differences (within a bRo5 subclass).

**Figure 6:**
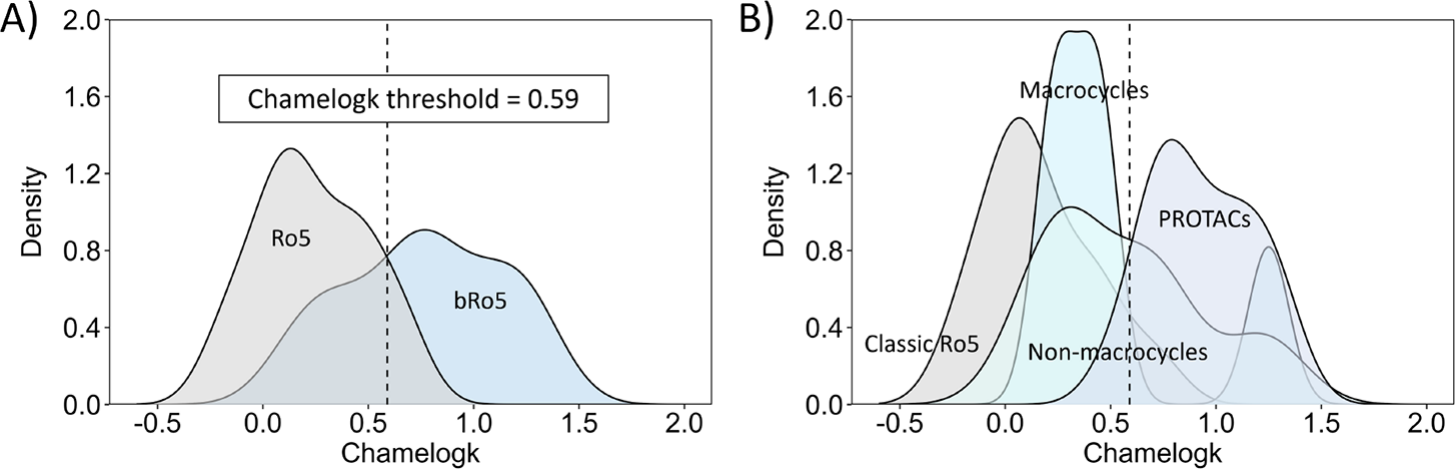
(A) Chamelogk density distribution for neutral Ro5 (*n* = 25) and bRo5 compounds (*n* = 30). (B) Chamelogk density distribution for bRo5 subclasses (*n* = 29): macrocycle (*n* = 5), nonmacrocycle (*n* = 6), and PROTAC (*n* = 18). For comparative purposes, only classical Ro5 compounds were displayed (*n* = 12) (E3 ligands and warheads were removed). Color coding is maintained with respect to Figure 2.

Therefore, with the aid of a density plot, we extracted the intersection point between the bRo5 and Ro5 density regions, defined at 0.59 (Figure 6A). Moreover, the defined threshold was applied to the density plot of bRo5 subclasses (Figure 6B), and we observed how the previously defined threshold is able to discard most macrocycles and preserve half of nonmacrocyclic drugs and most PROTACs. Overall, this fact supports the selection of 0.6 as a general alert threshold of chameleonicity. However, we are aware that more data are needed for a conclusive subclass-based threshold definition.

### Chameleonicity in Practice

According to the literature, oral bioavailability is driven by solubility, permeability, and metabolism (the latter is beyond the aim of this study).^48,49^ However, high-quality solubility and permeability data are not trivial to obtain for bRo5 derivatives.^50^ For instance, kinetic and thermodynamic solubilities are not often correlated, and the role played by active transport in permeability processes is poorly understood.^11,51^ Moreover, for most PROTACs, both solubility and permeability experiments can be affected by the sticky properties of the compounds. Therefore, the use of *ad hoc* chromatographic descriptors to provide a first screening of the solubility/permeability profile of drug candidates and thus reduce the number of solubility and permeability measurements could improve the efficiency of the drug discovery bRo5 pipeline.^11,23,44,51^ To highlight the relevance of this strategy, we recently used BRlogD and log *k*_w_^IAM^ (lipophilicity) to classify the solubility of 15 unrelated PROTACs^44^ and Δ log *k*_W_^IAM^ (polarity) to model cellular passive permeability^41^ for a reduced set of PROTACs. Moreover, another polarity descriptor, EPSA, is widely implemented in drug discovery to classify cell permeability of cyclic peptides.^30^ Data reported here showed that Chamelogk is not correlated to BRlogD and Δ log *k*_W_^IAM^ (Figure S3). Therefore, we were not surprised that, for the investigated compounds, Chamelogk is not correlated with thermodynamic solubility and permeability (not shown). However, as detailed below, chameleonicity can explain the oral bioavailability of drugs, showing either a too low solubility or a too low permeability. According to our experience, a compound with BRlogD >5 is too lipophilic, whereas one with Δ log *k*_W_^IAM^ > 1.5 is too polar for showing an acceptable solubility/permeability profile. In both cases, a chameleonic behavior could help compensate for these undesired values, and in practice, compounds having a Chamelogk >0.6 may exhibit this skill. Afterward, we retrieved several examples of well-known bRo5 compounds or drugs that may take advantage of chameleonicity to be oral and thus support our hypotheses.

**Figure 7:**
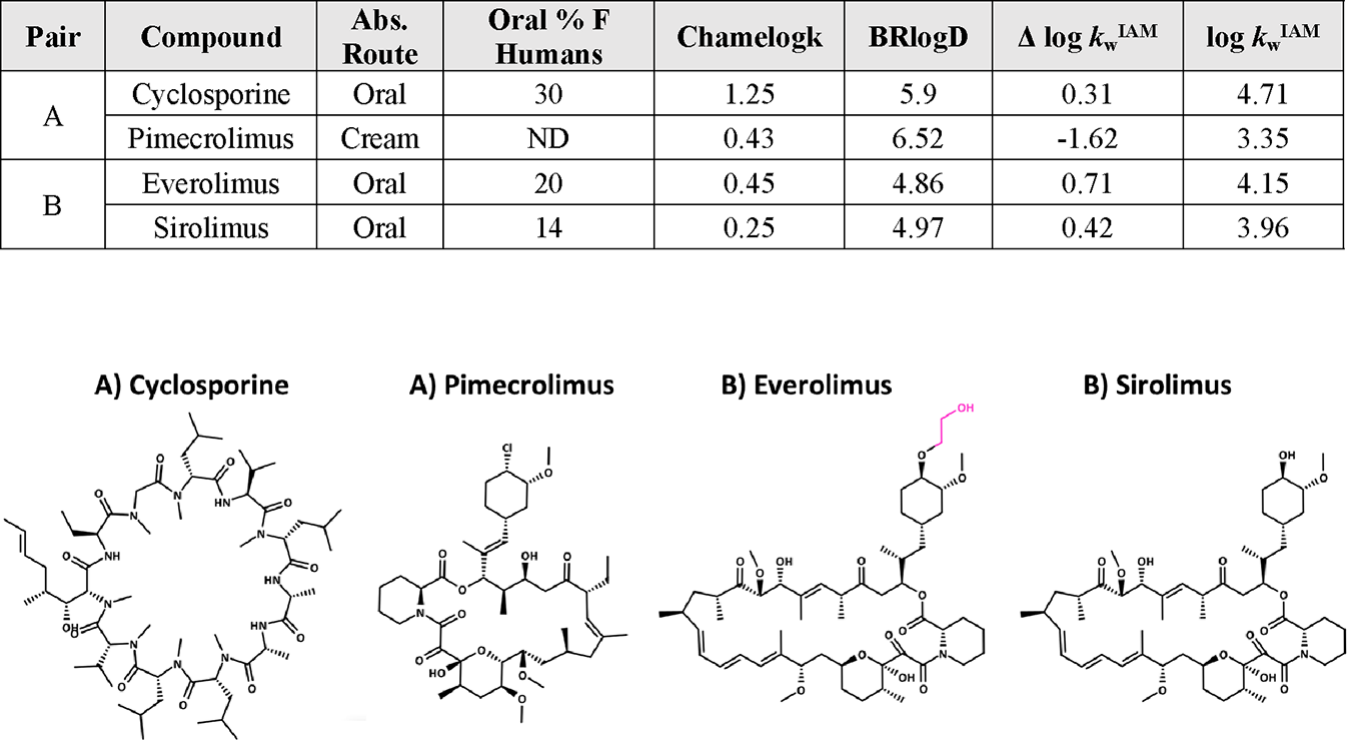
Macrocyclic drugs and their molecular properties. Abs.: absorption.

First, we address macrocycles (MCs). Our MC data set suggests that they are generally very lipophilic and poorly polar. Among the FDA-approved MC drugs, we focus on cyclosporine, an oral drug, and pimecrolimus, a cream (Figure 7). Both compounds have an extremely high BRlogD and low Δ log *k*_W_^IAM^, which are expected to provide high membrane retention and low solubility. Pimecrolimus has a low Chamelogk value (0.43), and thus, chameleonicity cannot compensate for the poor solubility. As a result, despite the proven oral bioavailability,^52^ pimecrolimus is used as a topical cream. Cyclosporine, on the other hand, is administered orally. We believe that the underlying reason of this behavior is chameleonicity, which allows CsA to adopt an open conformation in water and become water-soluble enough to be dosed orally (aqueous solubility at 25 °C = 23 μM).^53^ Chameleonicity, however, is not always needed to obtain an oral macrocyclic drug. For instance, sirolimus and everolimus^54^ (pharmacokinetically improved version of sirolimus with better bioavailability^1^) have an adequate lipophilicity/polarity balance (Figure 7), and thus, a chameleonic behavior is not necessary.

**Figure 8:**
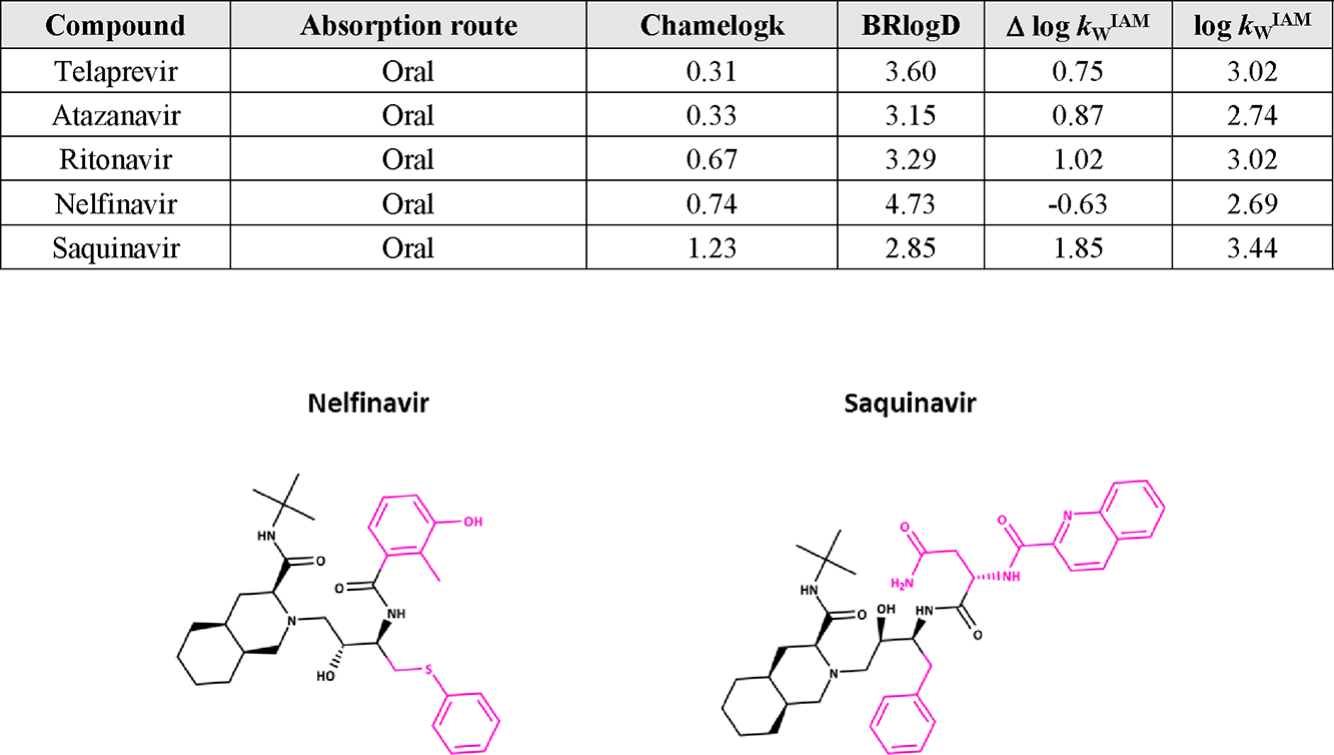
Nonmacrocyclic oral drugs and their molecular properties. The common atomic scaffold for saquinavir and nelfinavir is colored in black (different chiral centers).

Nonmacrocyclic bRo5 candidates also show wide ranges of chameleonicity (Figure 2B). Within our data set, we identified a few FDA-approved, orally absorbed, and structurally related antivirals: telaprevir, atazanavir, ritonavir, nelfinavir, and saquinavir. The first three already show similar and balanced BRlogD and Δ log *k*_W_^IAM^ values, suitable for oral absorption regardless of any chameleonicity contribution (Figure 8). Nelfinavir and saquinavir, on the other hand, are somehow different. Nelfinavir is more lipophilic than any other antiviral, but BRlogD is still below 5. In any case, it has an intermediate chameleonicity that could help to improve solubility. Conversely, saquinavir’s Δ log *k*_W_^IAM^ is extremely high (1.85) and considerably limits passive cell permeability.^27^ A low BRlogD is synonymous to acceptable solubility.^44^ Thus, the chameleonic properties of saquinavir (Chamelogk = 1.23) seem crucial to mask polarity^27^ and allow it to assume a less polar conformation in nonpolar media and hence to cross membranes (even if partly occurring by active transport).^27^

**Figure 9:**
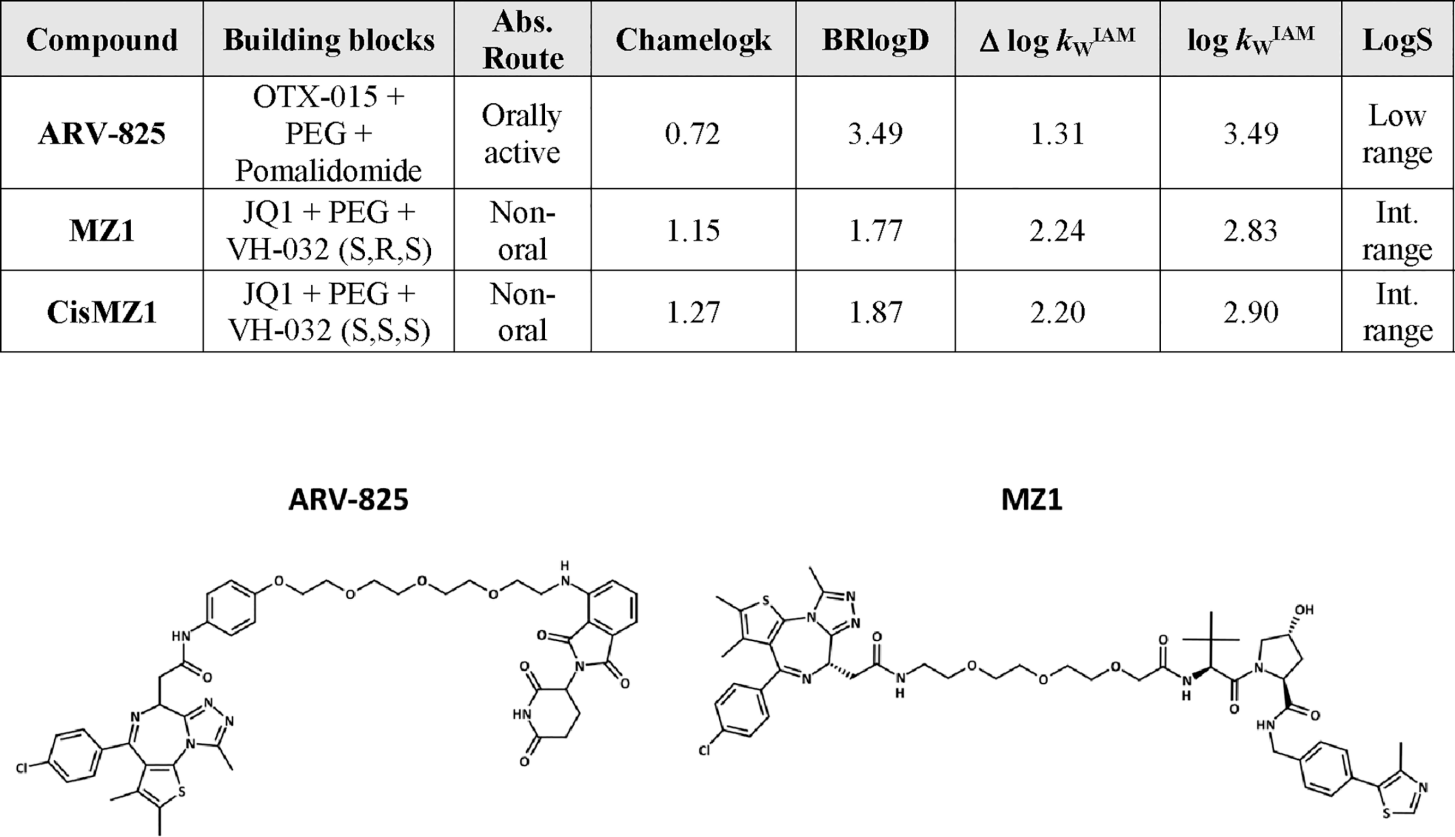
PROTACs and their molecular properties. Abs.: absorption; Int.: intermediate.

PROTACs occupy a completely different chemical space compared to other bRo5 drugs.^24^ Not only is the structure bigger and more flexible, but it occupies variable polarity and lipophilicity regions. According to the literature, many PROTACs suffer from solubility and/or permeability limitations, which have led to a low number of orally bioavailable PROTACs. In our data set, PROTACs are extremely polar (median Δ log *k*_W_^IAM^ = 2) and not so lipophilic (median BRlogD = 2.6, Table S5). Notably, the considered PROTACs are all chameleons (Figure 3B) regardless of the E3 ligase used. Even though CRBN allows the synthesis of more drug-like PROTACs,^55^ VHL-binding PROTACs can display higher chameleonicity (median values for VHL and CRBN PROTACs: 1.16 and 0.8, respectively). Within our data set, only ARV-825 has been proven to be orally active.^56^ ARV-825 is a CRBN-based PROTAC degrading the bromodomain-containing protein 4 (BRD4) (Figure 9) with an intermediate BRlogD (3.49) and a reasonably low polarity (1.31). Moreover, it also has a rather intermediate chameleonicity (0.72). Overall, ARV-825 has already an acceptable polarity and lipophilicity profile that can be slightly improved by chameleonicity. However, when compared to nonoral PROTACs, for example, MZ1, ARV-825 shows notable differences. MZ1 is a BRD4 selective PROTAC that uses a pegylated linker and a VHL-based E3 ligase.^57^ MZ1 is well-described in the literature and represents a good example of a nonoral PROTAC, as reported by opnMe^58^ (Boehringer Ingelheim). In terms of molecular properties, it is extremely polar (Δ log *k*_W_^IAM^ = 2.24) and poorly lipophilic (BRlogD = 1.77). Consequently, its permeability profile is poor (worse than ARV-825); thus, despite the high chameleonicity (Chamelogk = 1.15) and higher solubility,^44^ it is not orally bioavailable. Moreover, *cis*MZ1, the inactive version of MZ1, shows a similar profile to MZ1.

**Figure 10:**
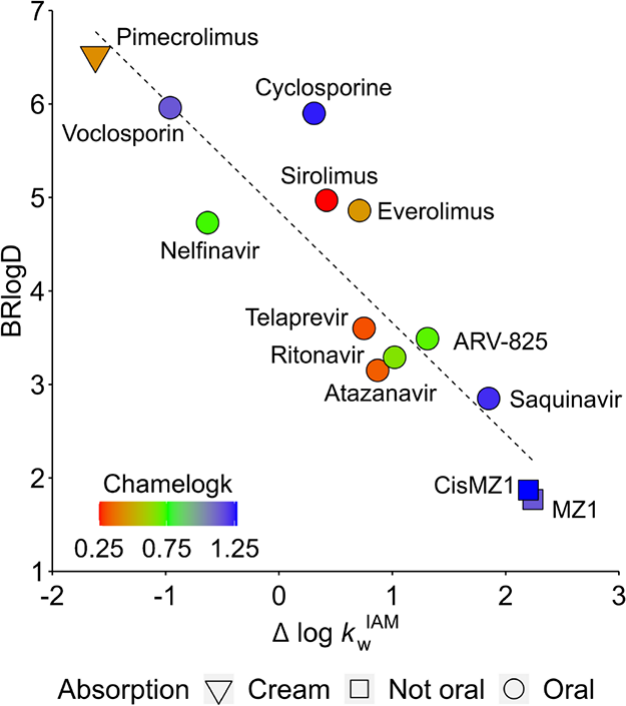
Polarity, lipophilicity, and chameleonicity representation for a set of bRo5 compounds. The absorption route is represented by different shapes (if approved). The dashed line represents the ideal linear slope for both variables.

Overall, our examples show that molecular chameleonicity can be useful when the lipophilicity/polarity balance is in a reasonable range to be corrected. Chamelogk (together with BRlogD and Δ log *k*_W_^IAM^) is a needed descriptor to check this opportunity. This is schematized in Figure 10, which supports that filling in an experimental property map for bRo5 candidates is the roadmap to improve the development of oral bRo5 drugs.

Obviously, we are interested in prospectively applying this approach to predict oral bioavailability from physicochemical descriptors. Here, we present a preliminary blind screening using voclosporin (a cyclosporine derivative recently approved by the FDA for the oral treatment of lupus nephritis, Figure S4) as an example. In practice, we tried to predict its oral bioavailability from lipophilicity, polarity, and chameleonicity descriptors. Data show that voclosporin is a strong chameleon (Chamelogk = 1.15) indeed able to correct the poor solubility of the compound as assessed by BRlogD (polarity is already in an acceptable range) (Figure 10).

## Conclusions

Chameleonicity is a molecular property of interest in the bRo5 chemical space. Here, we provide a fast chromatographic approach for its quantification. We hypothesized that by changing the polarity of the mobile phase, if a compound can display different conformational ensembles with different properties (*alias* a chameleon), the affinity for the column could variate in accordance to the properties of the compound rather than proportionally to the polarity of the system. Overall, Chamelogk is able to capture a property change relative to its original behavior (linear trend) that allows the direct comparison of extremely different structures. Chamelogk determination can be easily automated, and thus, it can be obtained in a high-throughput format. The evaluation of a larger amount of data, in due course in our laboratories (including proprietary compounds), will allow the refinement of the threshold to distinguish chameleons from nonchameleons now set at 0.6. Moreover, acidic and basic compounds fully ionized at pH = 7 are being currently investigated.

Chamelogk allowed us, for the first time, to propose a rationalization of the true impact of chameleonicity on the solubility/permeability balance and thus make easier oral bioavailability predictions in early drug discovery. Some selected examples of macrocycles, nonmacrocyclic compounds, and PROTACs revealed the role played by chameleonicity in adjusting a nonoptimal solubility/permeability balance, as highlighted by lipophilicity and polarity experimental descriptors.

Another application of Chamelogk will be the validation of computational attempts to characterize chameleonicity. We and other research groups are carrying out this effort obtaining up-to-now encouraging although not definitive results.

Overall, in this paper, we disclose and validate Chamelogk as a chameleonicity descriptor and provide the proof of concept that chameleonicity can be used in practice for bRo5 drug design. However, the impact of chameleonicity as an absolute molecular optimizer of ADME properties is still to be fully understood and is expected to be particularly relevant for prioritizing structurally related pairs.

## Materials and Methods

### Our Data Set

The 55 neutral compounds in our data set were purchased from several commercial sources or supplied by academic collaborations with pharmaceutical companies. The purities are provided in Table S6 and Figure S5.

### Solvents and Reagents

Ammonium acetate (CH_3_COONH_4_) was purchased from Alfa Aesar. In addition, HPLC-grade acetonitrile (MeCN) and methanol (MeOH) were purchased from VWR Chemicals. Milli-Q water was used in all experiments.

### Instruments

The HPLC DIONEX Ultimate 3000 (Thermo Scientific Inc.) coupled to an RS diode array and the Chromeleon 7.2.10 software (www. thermofisher.com) was used for all chromatographic measurements. The variety of experimental descriptors required specific chromatographic columns. Three different columns were used: IAM.PC.DD2 (300 Å, 10 μm, 10 cm × 4.6 mm) from REGIS, PLRP-S polymeric reversed-phase column (100 Å, 5 μm, 50 × 4.6 mm) from Agilent (www.agilent.com), and XBridge Shield RP18 (130 Å, 5 μm, 5 cm × 4.6 mm) from Waters (www.waters.com). High-performance ergonomic single-channel variable volume pipettors, 1.5 mL HPLC vials, and 9 mm PP screw caps were purchased from VWR Signature. The pH of each buffer and sample was controlled using a Eutech pH Meter 2700 (www.fishersci.com).

### Chromatographic Environments

The mobile phases for every descriptor consisted of isocratic solutions of 20 mM ammonium acetate at pH 7.0 and acetonitrile at various percentages (see the specific descriptor). Small amounts of the 55 compounds were dissolved in buffer/acetonitrile mixtures (v/v) at concentrations ranging from 50 to 100 μg/mL. Subsequently, 10 μL of each solution (injection volume) was injected at an isocratic flow rate of 1 mL/min and analyzed at 30 °C (oven temperature). Chromatographic measurements were then analyzed in duplicate based on the specific chromatographic conditions of each descriptor.

#### The PLRP-S System

We measured the RT of every compound in the data set at six mobile phase conditions (50 to 100% MeCN) using the PLRP-S column.^40^ Next, we calculated the capacity factor (log k’ PLRP-S) using eq 2:
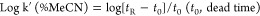
2and plotted it at each mobile phase composition (% MeCN) (see Figure 1, S1). Chamelogk: to quantify chameleonicity, the mobile phase conditions that respected a linear behavior for bRo5 compounds (50, 60, and 70% MeCN) were selected to build a linear trend. Compounds with a linear *R*^2^ lower than 0.8 were discarded. In addition, 100% MeCN was selected as the mobile phase condition with the highest capacity factor change and chosen for comparison and Chamelogk calculation (eq 1). Moreover, acetone, caffeine, phenol and a mixture of uracile, acetophenone and toluene were used as gold standards.

#### The XBridge System

BRlogD^39^ required the injection of 55 samples into the X-Bridge column at 60% MeCN (predominant mobile phase constituent). The retention times and dead time (*t*_0_, baseline interference) were recorded, and the capacity factor log k’60 was calculated adapting eq 2. Lastly, BRlogD value was calculated from eq 3
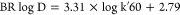
3

In this case, BRlogD required the measurement of acetone, caffeine, ibuprofen, lidocaine, phenol, and a mixture of uracile, acetophenone, and toluene as gold standards.

#### The IAM (Immobilized Artificial Membrane) System

Log *k*_W_^IAM^ involved the dissolution and injection of the samples into the IAM column at different mobile phases (from 10 to 50% MeCN). The retention times of the samples were recorded, and the capacity factor was calculated for each mobile phase condition adapting eq 2, where *t*_0_ is the retention time of citric acid. Besides, five standards (caffeine, carbamazepine, ketoprofen, theobromine, and toluene) were examined on a daily basis. Finally, the log *k*_W_^IAM^ value for each compound was calculated by extrapolating from the equation obtained with the five mobile phase conditions (10 to 50% MeCN) the capacity factor at a completely aqueous environment (100% buffer/0% MeCN).^45^ Δ log *k*_W_^IAM^ was previously defined by Grumetto et al.^46,47^ as
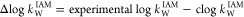
4with clog *k*_W_^IAM^ being the log *k*_W_^IAM^ value for nonpolar and neutral compounds^47^ with PSA = 0. Moreover, clog *k*_W_^IAM^ was correlated with the log P value (octanol/water)^47^ and afterward with the chromatographic descriptor BRlogD using eq 5:^39^
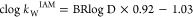
5Thus, Δ log *k*_W_^IAM^ requires the measurement of BRlogD and log *k*_W_^IAM^.

### Molecular Descriptors

The simplified molecular input line-entry system (SMILES) codes of the 55 compounds were downloaded from Pubchem (www.pubchem.com), and their 2D molecular properties were calculated. NAR was calculated with OSIRIS DataWarrior (http://www.openmolecules.org/datawarrior/, version 5.5.0, 2021),^60^ and the molecular weight (MW), number of carbon atoms (nC), topological polar surface area (TPSA), number of hydrogen bond acceptors (HBA) and donors (HBD), and Kier flexibility index (Φ or PHI) were calculated using the Dragon software (Kode srl, software for molecular descriptor calculation, https://chm.kode-solutions.net/pf/dragon-7-0/, version 7.0.10, 2017) and AlvaDesc (Alvascience, Software for Molecular Descriptors Calculation, www.alvascience.com/alvadesc/, ver. 1.0.18, 2020). Moreover, the number of HBDs was calculated by adding up the hydrogen atoms adjacent to any nitrogen and oxygen without negative charge in the molecule. HBAs were calculated as the total sum of nitrogen, oxygen, and fluorine atoms. Nevertheless, nitrogen atoms with positive formal charges, higher oxidation states, or bearing pyrrolyl forms were not considered as HBA. The neutral state as pH 7 was verified with Marvinsketch (ChemAxon, https://www.chemaxon.com, ver. 22.13.02022).

### Statistical and ML Analysis

All plots and analyses were performed with GraphPad Prism version 9.0 (www.graphpad.com) and RStudio (version 2022.02.3, package ggplot). Statistical tests were performed using the nonparametric Wilcoxon test. To define an indicative chameleonicity threshold, the density curve for Chamelogk was plotted. Next, the intersection between the Ro5 and bRo5 distributions was defined, and the chameleonicity threshold was plotted on the bRo5 subclass density plot for comparison.

## Supporting Information Available

Calculated 2D descriptors for acetophenone and cyclosporine; calculated 2D descriptors for the complete data set; 2D descriptors of pomalidomide (CRBN binder) and VHL-032 (VHL binder); E3-binder classification for the E3 ligand and PROTAC subclasses; experimental polarity, lipophilicity, and chameleonicity for PROTACs; purity and experimental wavelength for the compound data set ; chameleonicity equivalence between Chamelogk and Δ 3D-PSA; correlation between Chamelogk and relevant experimental polarity and lipophilicity descriptors; and HPLC purity assessment for the studied compounds (PDF)
Formula strings are provided as SMILES (CSV)

## Abbreviations Used

bRo5: beyond the rule of 5
CRBN: cereblon
CsA: cyclosporine A
CSD: Cambridge Structural Database
IAM: immobilized artificial membrane
IMHBs: intramolecular hydrogen bonds
MC: macrocycles
MeCN: acetonitrile
NAMFIS: NMR analysis of molecular flexibility in solution
NAR: number of aromatic rings
nC: number of carbon atoms
PHI or Φ: Kier’s flexibility index
PLRP: polymeric reversed-phase column
POI: protein of interest
PROTAC: proteolysis targeting chimera
RGyr: radius of gyration
RP: reverse-phase
TPSA: topological polar surface area
SMILES: simplified molecular input line-entry system
VHL: Von Hippel–Lindau
