# Supplementary material for: Chamelogk: A Chromatographic Chameleonicity Quantifier to Design Orally Bioavailable Beyond-Rule-of-5 Drugs
Source: J Med Chem. 2023 Jul 25;66(15):10681–93. doi: 10.1021/acs.jmedchem.3c00823 (PMC10424176; doi:10.1021/acs.jmedchem.3c00823)
Supplement: Supplementary file 1 — jm3c00823_si_001.pdf [file jm3c00823_si_001.pdf]

Supporting Information

Chamelogk: a chromatographic chameleonicity quantifier to design orally bioavailable beyond-Rule-of-5 drugs

Diego Garcia Jimenez, Maura Vallaro, Matteo Rossi Sebastiano, Giulia Apprato, Giulia D'Agostini, Paolo Rossetti, Giuseppe Ermondi and Giulia Caron*

University of Torino, Molecular Biotechnology and Health Sciences Dept., CASSMedChem, via Quarello 15, 10135 Torino, Italy.

Corresponding author*: Giulia Caron -Email: giulia.caron@unito.it

List of contents

Supplementary csv file: Supporting information containing the SMILES strings for the studied dataset.

Supplementary tables:

- Supplementary Table 1: *calculated 2D descriptors for Acetophenone and Cyclosporine.*

- Supplementary Table 2: *calculated 2D descriptors for the complete dataset.*

- Supplementary Table 3: *2D descriptors of pomalidomide (CRBN binder) and VHL-032 (VHL binder).*

- Supplementary Table 4: *E3-binder classification for the E3 ligand and PROTAC subclasses*

- Supplementary Table 5: *experimental polarity, lipophilicity and chameleonicity for PROTACs.*

- Supplementary Table 6: *purity and experimental wavelength for all the compounds.*

Supplementary figures:

- Supplementary Figure 1: *log k´variation of Ro5 and bRo5 compounds in the PLRP-S system.*

- Supplementary Figure 2: *chameleonicity equivalence between Chamelogk and Δ 3D-PSA.*

- Supplementary Figure 3: *correlation between Chamelogk and relevant experimental polarity and lipophilicity descriptors.*

- Supplementary Figure 4: *Voclosporin and its experimental descriptors.*

- Supplementary Figure 5: *HPLC purity assessment for the studied compounds (table S6)*

Table S1*. Calculated 2D descriptors for Acetophenone and Cyclosporine.*

Compound	Ro5/bRo5	Chamelogk	MW	nC	HBD	HBA	TPSA	PHI
**Acetophenone**	Ro5	-0.04	120.2	8	0	1	17.1	1.6
**Cyclosporine**	bRo5	1.25	1202.9	62	5	23	278.8	33.6

Table S2. *Calculated 2D descriptors for the complete neutral dataset. The corresponding SMILES codes are given as a supplementary CSV file.*

Compound	Class	Subclass	Chamelogk	MW	nC	HBD	HBA	TPSA	PHI	NRtB	NAR
**3-Bromoquinoline**	Ro5	Classic Ro5	0.13	208.06	9	0	1	12.89	1.63	0	2
**Acetone**	Ro5	Classic Ro5	-0.13	58.09	3	0	1	17.07	0.96	0	0
**Acetophenone**	Ro5	Classic Ro5	-0.04	120.16	8	0	1	17.07	1.64	1	1
**Bifonazole**	Ro5	Classic Ro5	0.45	310.42	22	0	1	17.82	4.05	4	4
**Clotrimazole**	Ro5	Classic Ro5	0.71	344.86	22	0	1	17.82	4.23	4	4
**Diazepam**	Ro5	Classic Ro5	0.3	284.76	16	0	3	32.67	3.39	1	2
**Diethylstilbestrol**	Ro5	Classic Ro5	0.44	268.38	18	2	2	40.46	4.72	4	2
**Hydrochlorothiazide**	Ro5	Classic Ro5	-0.22	297.77	7	4	7	135.12	2.94	1	1
**Hydrocortisone**	Ro5	Classic Ro5	0.1	362.51	21	3	5	94.83	4.05	2	0
**Naphthalene**	Ro5	Classic Ro5	0.02	128.18	10	0	0	0	1.18	0	2
**Phenol**	Ro5	Classic Ro5	0.16	94.12	6	1	1	20.23	1.09	0	1
**Toluene**	Ro5	Classic Ro5	0.06	92.15	7	0	0	0	1.12	0	1
**4-F-thalidomide**	Ro5	E3 Ligand	0.23	276.24	13	1	7	85.24	2.82	1	1
**4-Hydroxy thalidomide**	Ro5	E3 Ligand	-0.16	274.25	13	2	7	105.47	2.84	1	1
**Cis-OH-VH298 (S,S,S)**	Ro5	E3 Ligand	0.45	539.72	27	4	10	183.89	8.07	8	2
**Cis-phenol-VH032 (S,S,S)**	Ro5	E3 Ligand	0.65	488.67	24	4	9	160.1	8.25	7	2
**OH-VH298 (S,R,S)**	Ro5	E3 Ligand	0.48	539.72	27	4	10	183.89	8.07	8	2
**Phenol-VH032 (S,R,S)**	Ro5	E3 Ligand	0.37	488.67	24	4	9	160.1	8.25	7	2
**Pomalidomide**	Ro5	E3 Ligand	0	273.27	13	3	7	111.26	2.84	1	1
**BI-0115**	Ro5	Warhead	0.19	287.77	15	1	4	50.68	3.53	2	2
**BI-1580**	Ro5	Warhead	0.1	253.33	15	1	4	50.68	2.9	1	2
**CPI203**	Ro5	Warhead	0.54	399.94	19	2	5	114.4	4.65	3	3
**HJB97**	Ro5	Warhead	0.15	500.62	26	3	9	135.78	5.93	7	5
**MS-417**	Ro5	Warhead	0.29	414.95	20	0	5	97.61	5.11	4	3
**OTX-015**	Ro5	Warhead	0.64	492.04	25	2	6	120.64	6.11	4	4
**Cyclosporine**	bRo5	Macrocycle	1.25	1202.84	62	5	23	278.8	33.57	15	0
**Everolimus**	bRo5	Macrocycle	0.45	958.37	53	3	15	204.66	23.29	9	0
**Pimecrolimus**	bRo5	Macrocycle	0.43	811	43	2	12	158	17	6	0
**Sirolimus**	bRo5	Macrocycle	0.25	914.31	51	3	14	195.43	21.32	6	0
**Temsirolimus**	bRo5	Macrocycle	0.23	1030.44	56	4	17	241.96	24.38	11	0
**Atazanavir**	bRo5	Non-macrocycle	0.33	704.96	38	5	13	171.22	15.43	18	3
**Nelfinavir**	bRo5	Non-macrocycle	0.74	567.87	32	4	7	127.2	10.61	10	2
**Paclitaxel**	bRo5	Non-macrocycle	0.15	853.99	47	4	15	221.29	12.38	14	3

**Ritonavir**	bRo5	Non-macrocycle	0.67	721.05	37	4	11	202.26	15.8	18	4
**Saquinavir**	bRo5	Non-macrocycle	1.23	670.94	38	6	11	166.75	12.22	13	3
**Telaprevir**	bRo5	Non-macrocycle	0.31	679.96	36	4	13	179.56	12.39	14	1
**PEG_4-PH-NH_2-Pomalidomide**	bRo5	E3 Ligand	0.12	540.63	27	4	12	160.21	10.56	15	2
**ARV-825**	bRo5	PROTAC	0.72	923.53	46	3	16	234.6	14.97	19	5
**BI-0319**	bRo5	PROTAC	0.99	1061.26	52	5	22	270	19.66	25	5
**BI-3663**	bRo5	PROTAC	0.5	917.93	44	4	22	244.47	15.56	21	4
**BI-4206**	bRo5	PROTAC	0.7	1061.26	52	5	22	270	19.66	25	5
**BRD4 degrader AT1**	bRo5	PROTAC	1.26	972.81	48	4	13	265.58	16.74	18	5
**CisMZ1**	bRo5	PROTAC	1.27	1002.77	49	4	16	267.97	18.07	21	5
**CRBN-6-5-5-VHL**	bRo5	PROTAC	1.05	972.34	51	5	17	255.6	20.44	27	3
**dBET1**	bRo5	PROTAC	0.8	785.35	38	3	14	223.98	11.42	12	4
**dBET57**	bRo5	PROTAC	0.68	699.25	34	3	12	197.68	8.99	8	4
**dBET6**	bRo5	PROTAC	0.86	841.47	42	3	14	223.98	13.52	16	4
**Gefitinib-based PROTAC 3**	bRo5	PROTAC	1.36	934.63	47	4	16	214.6	18.44	23	5
**MZ1**	bRo5	PROTAC	1.15	1002.77	49	4	16	267.97	18.07	21	5
**MZP-54**	bRo5	PROTAC	1.18	1036.8	55	5	16	229	19.77	22	5
**PROTAC BET Degrader-10**	bRo5	PROTAC	0.83	783.38	39	3	13	214.75	11.45	12	4
**PROTAC FAK degrader 1**	bRo5	PROTAC	0.79	996.26	47	5	20	253.93	17.97	21	5
**PROTAC Mcl degrader-1**	bRo5	PROTAC	0.93	909.93	45	4	14	219.84	14.74	19	4
**PROTAC-1**	bRo5	PROTAC	1.07	1034.24	50	4	22	264.87	19.05	22	5
**ZXH-3-26**	bRo5	PROTAC	0.65	785.35	38	3	14	223.98	11.42	13	4

Table S3*. 2D descriptors of pomalidomide (CRBN binder) and VHL-032 (VHL binder). S,R,S-AHPC HCl was selected as the amine derivative of VHL-032.*

E3 ligand	Chamelogk	Charge	MW	nC	HBD	HBA	TPSA	PHI
**Pomalidomide**	0	Neutral	273.3	13	3	7	111.3	2.8
**VHL-032 (S,R,S-AHPC HCl)**	2.12	+	430.6	22	4	7	136.8	10.6

Table S4. *E3-binder classification for the E3 ligand and PROTAC subclasses.*

Compound	Class	Subclass	E3 ligase	Chamelogk
**BI-1580**	Ro5	Warhead		0.10
**HJB97**	Ro5	Warhead		0.15
**BI-0115**	Ro5	Warhead		0.19
**MS-417**	Ro5	Warhead		0.29
**CPI203**	Ro5	Warhead		0.54
**OTX-015**	Ro5	Warhead		0.64
**4-Hydroxy thalidomide**	Ro5	Ligand	CRBN	-0.16
**Pomalidomide**	Ro5	Ligand	CRBN	0.00
**4-F-thalidomide**	Ro5	Ligand	CRBN	0.23
**Phenol-VH032 (S,R,S)**	Ro5	Ligand	VHL	0.37
**Cis-OH-VH298 (S,S,S)**	Ro5	Ligand	VHL	0.45
**OH-VH298 (S,R,S)**	Ro5	Ligand	VHL	0.48
**Cis-phenol-VH032 (S,S,S)**	Ro5	Ligand	VHL	0.65
**PEG_4-PH-NH_2-Pomalidomide**	bRo5	Ligand	CRBN	0.12
**BI-3663**	bRo5	PROTAC	CRBN	0.50
**ZXH-3-26**	bRo5	PROTAC	CRBN	0.65
**dBET57**	bRo5	PROTAC	CRBN	0.68
**ARV-825**	bRo5	PROTAC	CRBN	0.72
**dBET1**	bRo5	PROTAC	CRBN	0.80
**PROTAC BET Degrader-10**	bRo5	PROTAC	CRBN	0.83
**dBET6**	bRo5	PROTAC	CRBN	0.86
**PROTAC Mcl degrader-1**	bRo5	PROTAC	CRBN	0.93
**CRBN-6-5-5-VHL**	bRo5	PROTAC	CRBN	1.05
**BI-4206**	bRo5	PROTAC	VHL	0.70
**PROTAC FAK degrader 1**	bRo5	PROTAC	VHL	0.79
**BI-0319**	bRo5	PROTAC	VHL	0.99
**PROTAC-1**	bRo5	PROTAC	VHL	1.07
**MZ1**	bRo5	PROTAC	VHL	1.15
**MZP-54**	bRo5	PROTAC	VHL	1.18
**BRD4 degrader AT1**	bRo5	PROTAC	VHL	1.26
**CisMZ1**	bRo5	PROTAC	VHL	1.27
**Gefitinib-based PROTAC 3**	bRo5	PROTAC	VHL	1.36

Table S5. *Experimental polarity, lipophilicity and chameleonicity for PROTACs.*

**PROTAC statistics**	**Chamelogk**	**BRlogD**	**Δ log k_W^{IAM}**
**Min**	0.5	1.5	0.2
**1st Quartile**	0.7	1.9	1.3
**Median**	**0.9**	**2.6**	**2.0**
**Mean**	0.9	2.5	1.8
**3rd Quartile**	1.1	2.9	2.2
**Max**	1.4	4.4	2.6

Table S6. *List of the studied compounds with their purity and measurement wavelength.*

Compound	Class	Subclass	Purity (%)	Wavelength (nm)
**Hydrochlorothiazide**	Ro5	Classic Ro5	99.8	225
**3-Bromoquinoline**	Ro5	Classic Ro5	97.7	309
**Acetone**	Ro5	Classic Ro5	96.9	254
**Acetophenone**	Ro5	Classic Ro5	96.7	254
**Bifonazole**	Ro5	Classic Ro5	98.8	254
**Clotrimazole**	Ro5	Classic Ro5	97.8	254
**Diazepam**	Ro5	Classic Ro5	95.6	254
**Diethylstilbestrol**	Ro5	Classic Ro5	99.7	225
**Hydrocortisone**	Ro5	Classic Ro5	96.8	254
**Naphthalene**	Ro5	Classic Ro5	98.9	254
**Phenol**	Ro5	Classic Ro5	97.7	225
**Toluene**	Ro5	Classic Ro5	99.6	225
**4-Hydroxy thalidomide**	Ro5	E3 Ligand	99.8	220
**4-F-thalidomide**	Ro5	E3 Ligand	92	213
**Cis-OH-VH298 (S,S,S)**	Ro5	E3 Ligand	92.5	206
**Cis-phenol-VH032 (S,S,S)**	Ro5	E3 Ligand	99.6	206
**OH-VH298 (S,R,S)**	Ro5	E3 Ligand	95.3	206
**Phenol-VH032 (S,R,S)**	Ro5	E3 Ligand	99.6	206
**Pomalidomide**	Ro5	E3 Ligand	99.8	225
**BI-0115**	Ro5	Warhead	96.2	254
**BI-1580**	Ro5	Warhead	95.4	254
**CPI203**	Ro5	Warhead	99.8	225
**HJB97**	Ro5	Warhead	98	225
**MS-417**	Ro5	Warhead	98.3	254
**OTX-015**	Ro5	Warhead	99.4	254
**Cyclosporine**	bRo5	Macrocycle	97.6	232
**Everolimus**	bRo5	Macrocycle	95.9	254
**Pimecrolimus**	bRo5	Macrocycle	90.9	225
**Sirolimus**	bRo5	Macrocycle	98.8	254
**Temsirolimus**	bRo5	Macrocycle	99.7	254
**Atazanavir**	bRo5	Non-macrocycle	95.2	254
**Nelfinavir**	bRo5	Non-macrocycle	98.7	254
**Paclitaxel**	bRo5	Non-macrocycle	95.3	254
**Ritonavir**	bRo5	Non-macrocycle	96.3	240
**Saquinavir**	bRo5	Non-macrocycle	96.3	254
**Telaprevir**	bRo5	Non-macrocycle	91.2	254
**PEG_4-PH-NH_2-Pomalidomide**	bRo5	E3 Ligand	93.2	284
**ARV-825**	bRo5	PROTAC	96.4	254
**BI-0319**	bRo5	PROTAC	97.1	254
**BI-3663**	bRo5	PROTAC	98.9	254
**BI-4206**	bRo5	PROTAC	95.5	254
**BRD4 degrader AT1**	bRo5	PROTAC	98	225
**CisMZ1**	bRo5	PROTAC	96.9	225
**CRBN-6-5-5-VHL**	bRo5	PROTAC	95.4	225
**dBET1**	bRo5	PROTAC	98.3	225
**dBET57**	bRo5	PROTAC	99.1	284
**dBET6**	bRo5	PROTAC	94.8	330
**Gefitinib-based PROTAC 3**	bRo5	PROTAC	99.6	254
**MZ1**	bRo5	PROTAC	99.6	254
**MZP-54**	bRo5	PROTAC	97.5	256
**PROTAC BET Degrader-10**	bRo5	PROTAC	97.7	225
**PROTAC FAK degrader 1**	bRo5	PROTAC	94.6	254
**PROTAC Mcl degrader-1**	bRo5	PROTAC	95.2	392
**PROTAC-1**	bRo5	PROTAC	93.6	225
**ZXH-3-26**	bRo5	PROTAC	96.3	225
**Voclosporine**	bRo5	Macrocycle	96.7	225

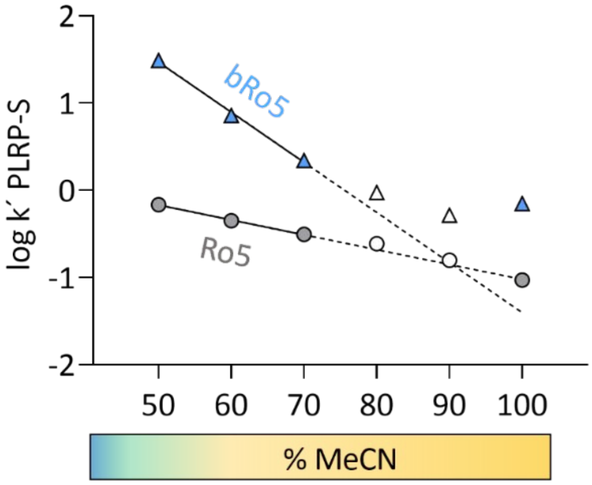

Figure S1. *Log k´variation of Ro5 (pomalidomide) and bRo5 (CsA) compounds in the PLRP-S system. The solid trend lines represent the equations obtained from experimental values at 50, 60 and 70% of MeCN. The dashed lines represent the extrapolation of the equation for higher MeCN percentages. White-colored symbols reflect the deviation from the equation.*

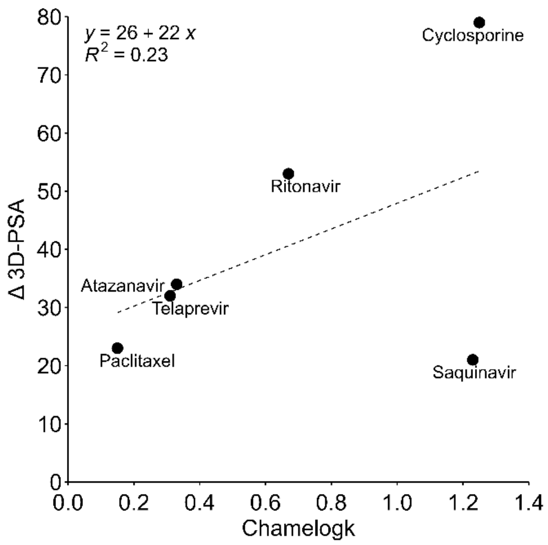

***Figure S2**. Chameleonicity equivalence between Chamelogk and Δ 3D-PSA. The dashed line represents the linear regression.*

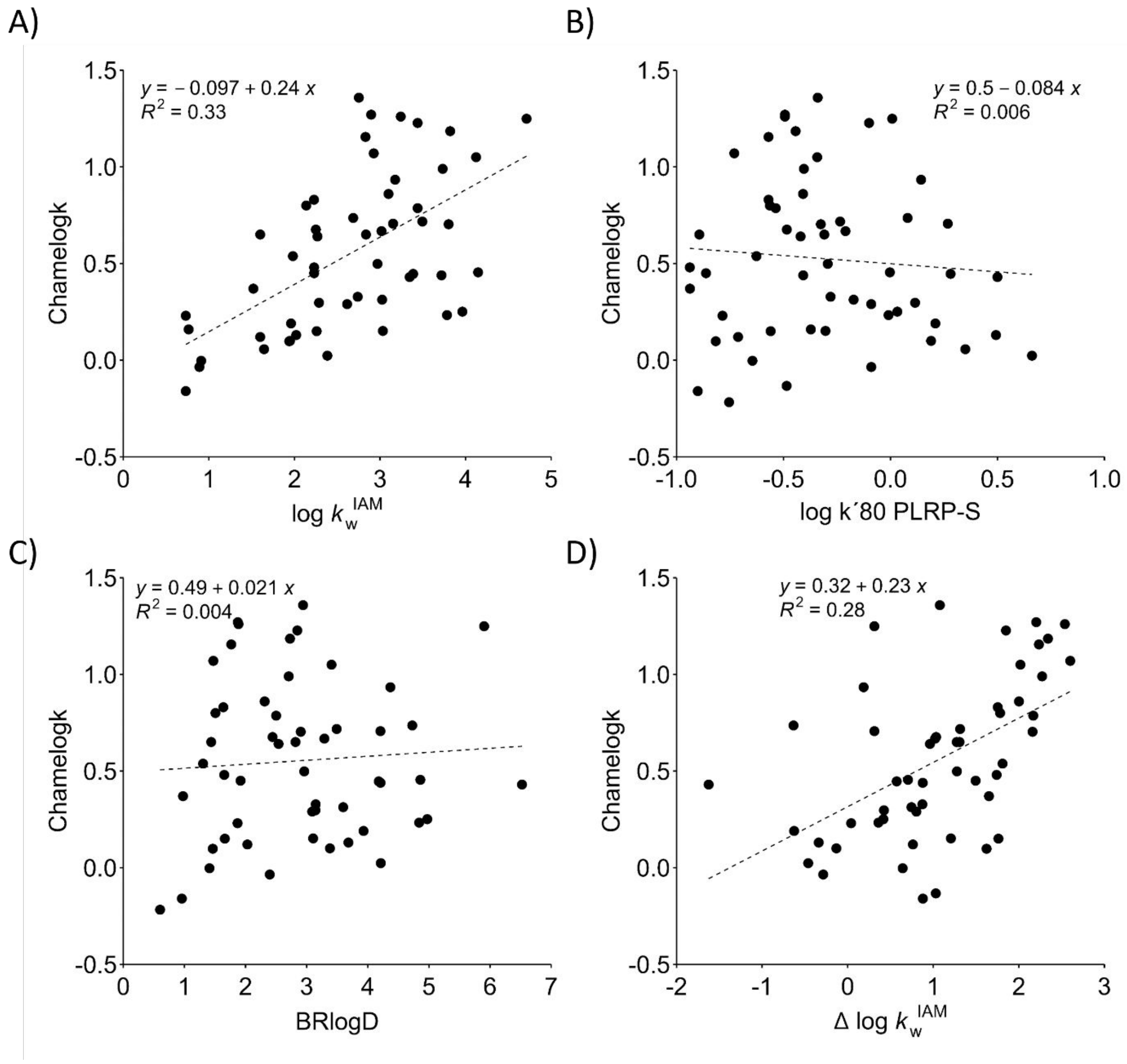

Figure S3. *Correlation between Chamelogk and relevant experimental polarity and lipophilicity descriptors; A) log* k_w *^{IAM} (n=53), B) log k´80 PLRP-S (n=55) , C) BRlogD (n=52) and D) Δ log* k_W *^{IAM} (n=52).*

A)

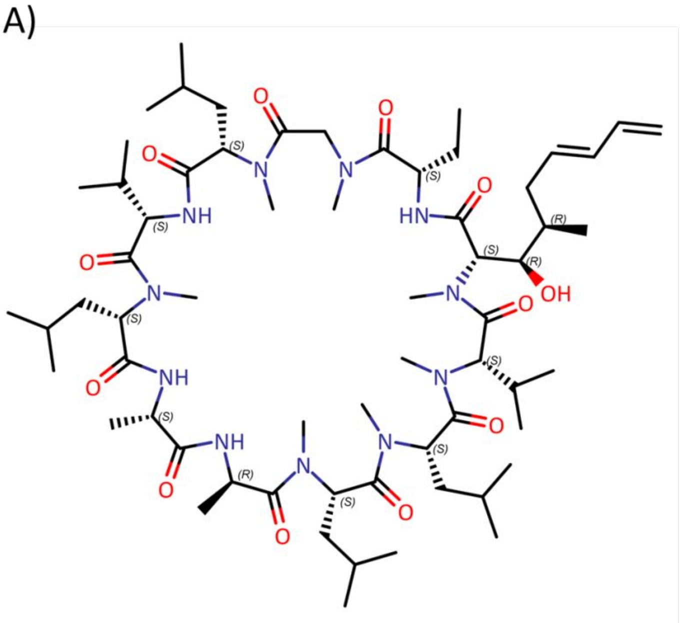

B)

Compound	Voclosporine
Class	bRo5
Subclass	Macrocycle
R^2	1.0
Chamelogk	1.15
BRlogD	5.96
log k_W^{IAM}	3.49
Δ log k_W^{IAM}	-0.96

Figure S4. *A) Voclosporin and B) its experimental descriptors.*

3-Bromoquinoline

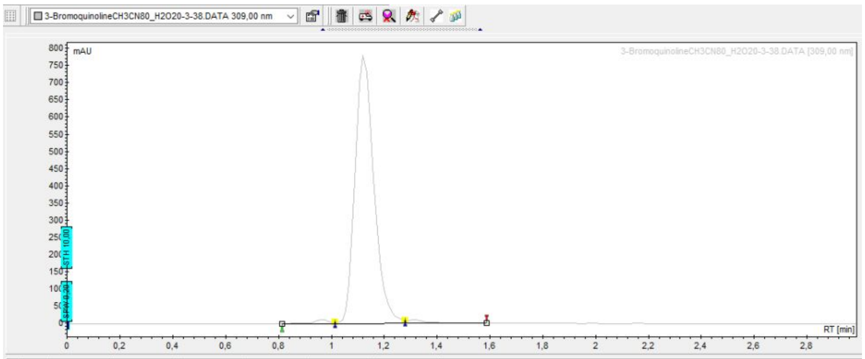

#	Name	Time [Min]	Quantity [% Area]	Height [mAU]	Area [mAU.Min]	Area % [%]
1	UNKNOWN	0,96	0,99	11,0	0,7	0,986
2	UNKNOWN	1,12	97,66	777,9	64,6	97,657
3	UNKNOWN	1,31	1,36	10,8	0,9	1,358
Total			100,00	799,7	66,2	100,000

Acetone

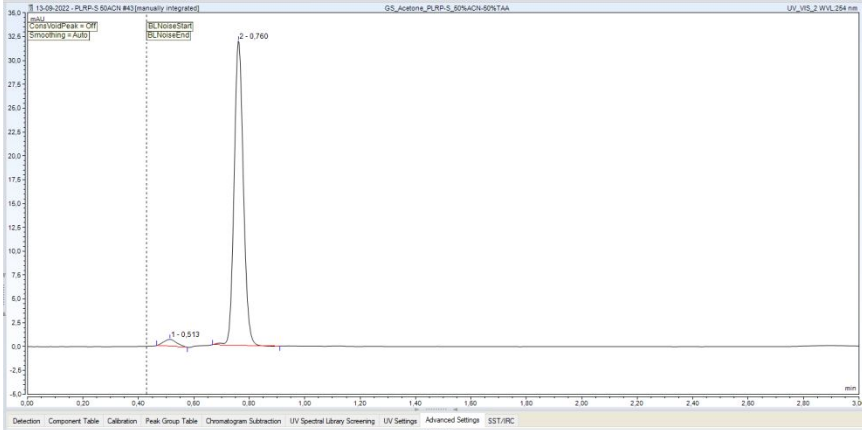

Peak No.	Peak Name	Ret.Time min	Amount n.a.	Rel.Area %	Area mAU*min	Height mAU	Type	Width (50%) min	Asym. EP	Resol. EP	Plates EP
1		0,513	n.a.	3,09	0,0392	0,73	BMB*	0,052	1,15	3,33	540
2		0,760	n.a.	96,91	1,2306	31,91	BMB*	0,035	1,15	n.a.	2540
Maximum			0,0000	96,91	1,2306	31,91		0,052	1,15	3,33	2540
Minimum			0,0000	3,09	0,0392	0,73		0,035	1,15	3,33	540
Sum			0,0000	100,00	1,2698	32,64					

Acetophenone

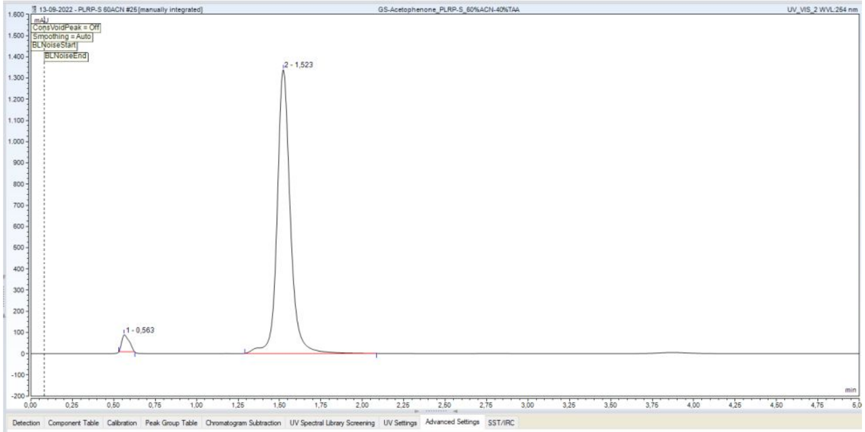

Peak No.	Peak Name	Ret.Time min	Amount n.a.	Rel.Area %	Area mAU*min	Height mAU	Type	Width (50%) min	Asym. EP	Resol. EP	Plates EP
1		0,563	n.a.	3,33	4,2388	81,36	BMB*	0,055	1,46	8,51	587
2		1,523	n.a.	96,67	123,1515	1337,08	BMB*	0,078	1,14	n.a.	2088
Maximum			0,0000	96,67	123,1515	1337,08		0,078	1,46	8,51	2088
Minimum			0,0000	3,33	4,2388	81,36		0,055	1,14	8,51	587
Sum			0,0000	100,00	127,3902	1418,43					

Bifonazole

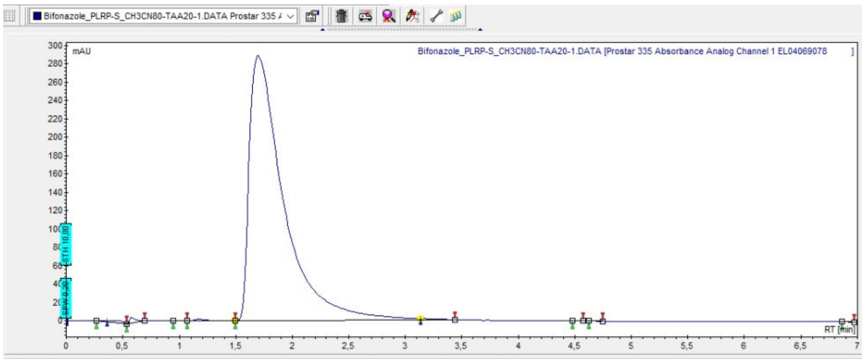

#	Name	Time [Min]	Quantity [% Area	Height [mAU]	Area [mAU.Min]	Area % [%]	Lipophilicity inde
1	UNKNOWN	0,36	0,05	1,0	0,1	0,050	-0,33
2	UNKNOWN	0,48	0,27	2,3	0,3	0,270	-0,10
3	UNKNOWN	0,57	0,36	6,0	0,4	0,356	0,07
4	UNKNOWN	1,01	0,01	0,2	0,0	0,011	0,90
5	UNKNOWN	1,17	0,29	1,9	0,3	0,291	1,20
6	UNKNOWN	1,69	98,81	288,7	101,8	98,814	2,17
7	UNKNOWN	3,16	0,19	1,4	0,2	0,186	4,92
8	UNKNOWN	4,53	0,00	0,1	0,0	0,004	7,49
9	UNKNOWN	4,65	0,01	0,1	0,0	0,007	7,71
10	UNKNOWN	6,93	0,01	0,2	0,0	0,011	11,98
Total			100,00	301,8	103,0	100,000	36,02

Clotrimazole

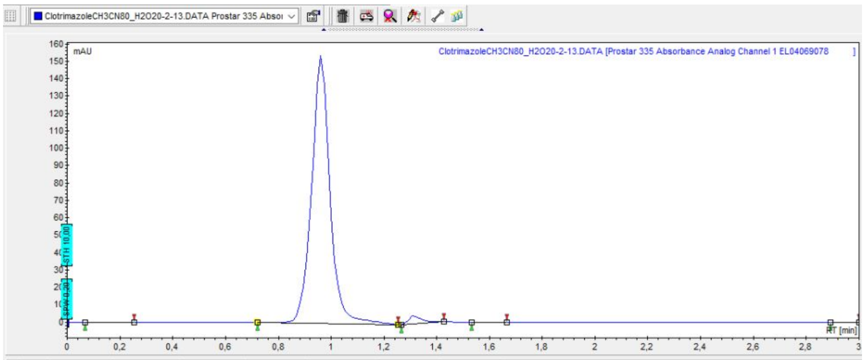

#	Name	Time [Min]	Quantity [% Area	Height [mAU]	Area [mAU.Min]	Area % [%]	Lipophilicity inde
1	UNKNOWN	0,17	0,09	0,1	0,0	0,090	-0,68
2	UNKNOWN	0,96	97,83	153,7	12,4	97,835	0,80
3	UNKNOWN	1,31	2,00	4,5	0,3	1,995	1,45
4	UNKNOWN	1,57	0,05	0,1	0,0	0,049	1,95
5	UNKNOWN	2,93	0,03	0,0	0,0	0,031	4,49
Total			100,00	158,5	12,6	100,000	8,01

Diazepam

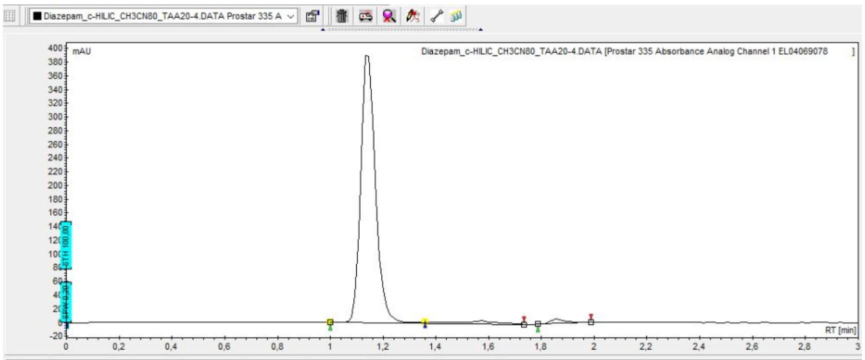

#	Name	Time [Min]	Quantity [% Area	Height [mAU]	Area [mAU.Min]	Area % [%]	Lipophilicity inde
1	UNKNOWN	1,13	95,64	389,5	25,5	95,645	1,12
2	UNKNOWN	1,57	2,68	4,3	0,7	2,684	1,95
3	UNKNOWN	1,85	1,67	6,9	0,4	1,671	2,47
Total			100,00	400,7	26,7	100,000	5,54

Diethylstilbestrol

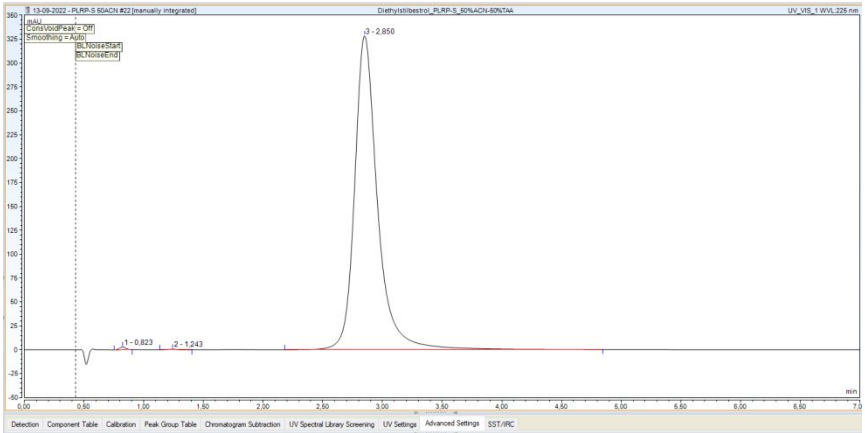

	A	B	C	D	E	F	G	H	I	J	K	L
1-2	Peak No.	Peak Name	Ret.Time min	Amount n.a.	Rel.Area %	Area mAU*min	Height mAU	Type	Width (50%) min	Asym. EP	Resol. EP	Plates EP
4	1		0,823	n.a.	0,24	0,1798	3,05	BMB*	0,056	1,06	4,51	1218
5	2		1,243	n.a.	0,05	0,0402	0,66	BMB*	0,054	0,86	7,83	2906
6	3		2,850	n.a.	99,70	73,2898	328,25	BMB*	0,188	1,31	n.a.	1273
7	Maximum			0,0000	99,70	73,2898	328,25		0,188	1,31	7,83	2906
8	Minimum			0,0000	0,05	0,0402	0,66		0,054	0,86	4,51	1218
9	Sum			0,0000	100,00	73,5098	331,96					

Hydrochlorothiazide

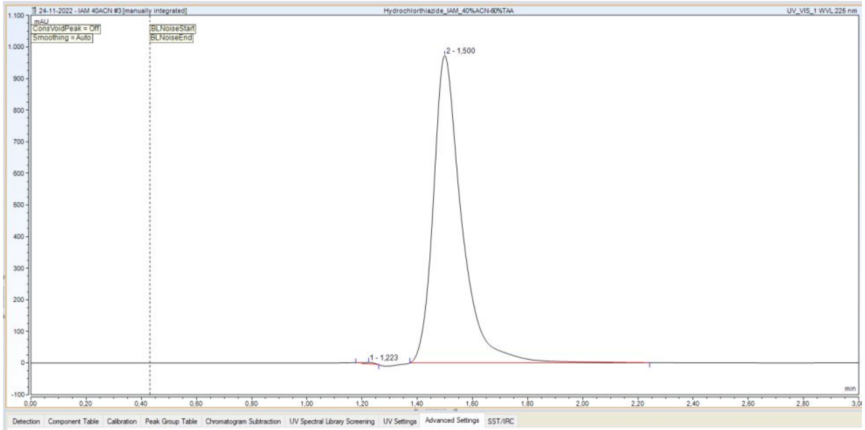

	A	B	C	D	E	F	G	H	I	J	K	L
1-2	Peak No.	Peak Name	Ret.Time min	Amount n.a.	Rel.Area %	Area mAU*min	Height mAU	Type	Width (50%) min	Asym. EP	Resol. EP	Plates EP
4	1		1,223	n.a.	0,17	0,1958	3,88	BMB*	0,049	0,91	2,19	3420
5	2		1,500	n.a.	99,83	115,4469	972,24	BMB*	0,100	1,42	n.a.	1244
6	Maximum			0,0000	99,83	115,4469	972,24		0,100	1,42	2,19	3420
7	Minimum			0,0000	0,17	0,1958	3,88		0,049	0,91	2,19	1244
8	Sum			0,0000	100,00	115,6427	976,12					

Hydrocortisone

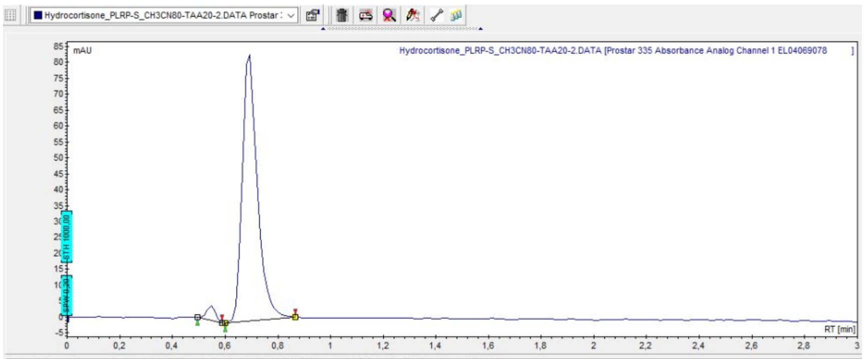

#	Name	Time [Min]	Quantity [% Area]	Height [mAU]	Area [mAU.Min]	Area % [%]	Lipophilicity inde
1	UNKNOWN	0,55	3,18	4,7	0,2	3,184	0,02
2	UNKNOWN	0,69	96,82	83,5	5,6	96,816	0,30
Total			100,00	88,3	5,8	100,000	0,32

Naphtalene

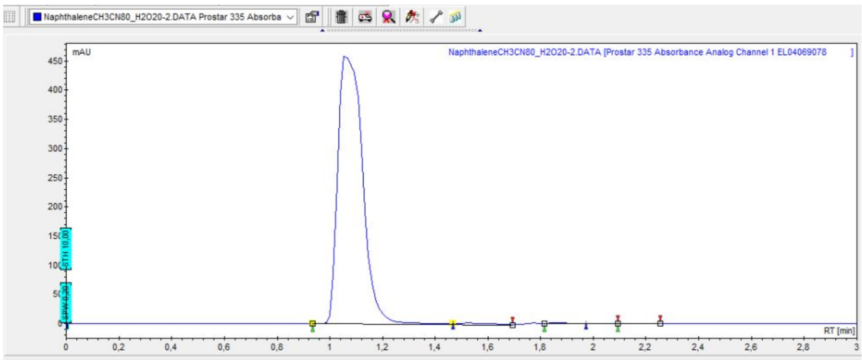

#	Name	Time [Min]	Quantity [% Area]	Height [mAU]	Area [mAU.Min]	Area % [%]	Lipophilicity inde
1	UNKNOWN	1,05	98,92	458,0	49,9	98,925	0,97
2	UNKNOWN	1,52	0,76	2,6	0,4	0,765	1,85
3	UNKNOWN	1,85	0,26	1,9	0,1	0,257	2,47
4	UNKNOWN	2,01	0,03	0,3	0,0	0,028	2,77
5	UNKNOWN	2,16	0,03	0,2	0,0	0,026	3,04
Total			100,00	463,0	50,4	100,000	11,10

Phenol

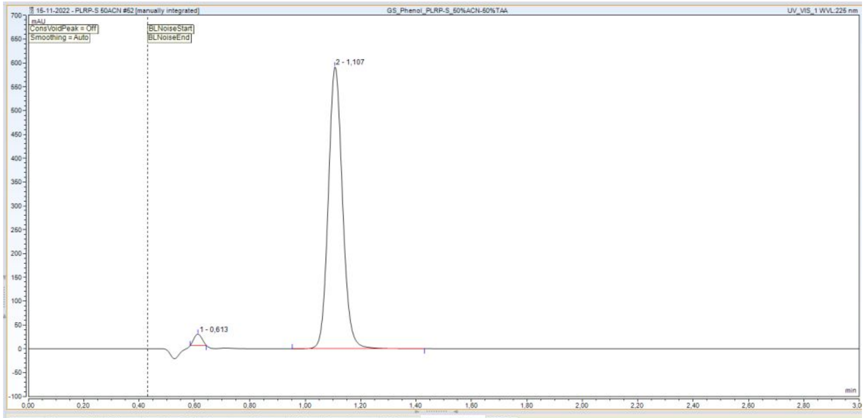

Detection | Component Table | Calibration | Peak Group Table | Chromatogram Subtraction | UV Spectral Library Screening | UV Settings | Advanced Settings | SST/IRC

Peak No.	Peak Name	Ret.Time min	Amount n.a.	Rel.Area %	Area mAU*min	Height mAU	Type	Width (50%) min	Asym. EP	Resol. EP	Plates EP
1		0,613	n.a.	2,28	0,8189	24,36	BMB*	0,034	0,99	6,62	1825
2		1,107	n.a.	97,72	35,1186	591,53	BMB*	0,054	1,13	n.a.	2315
Maximum			0,0000	97,72	35,1186	591,53		0,054	1,13	6,62	2315
Minimum			0,0000	2,28	0,8189	24,36		0,034	0,99	6,62	1825
Sum			0,0000	100,00	35,9375	615,89					

Toluene

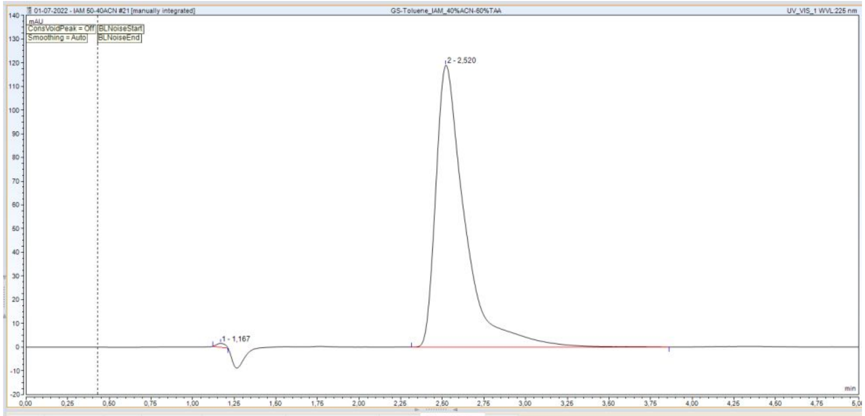

Peak No.	Peak Name	Ret.Time min	Amount n.a.	Rel.Area %	Area mAU*min	Height mAU	Type	Width (50%) min	Asym. EP	Resol. EP	Plates EP
1		1,167	n.a.	0,42	0,1024	1,62	BMB*	0,065	1,02	6,84	1787
2		2,520	n.a.	99,58	24,1061	118,96	BMB*	0,169	2,13	n.a.	1238
Maximum			0,0000	99,58	24,1061	118,96		0,169	2,13	6,84	1787
Minimum			0,0000	0,42	0,1024	1,62		0,065	1,02	6,84	1238
Sum			0,0000	100,00	24,2085	120,59					

4-F-thalidomide

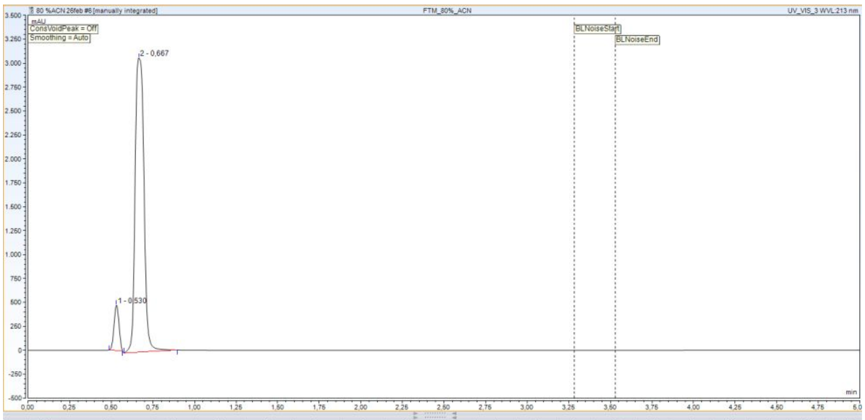

	A	B	C	D	E	F	G	H	I	J	K	L
1	Peak	Peak Name	Ret.Time	Amount	Rel.Area	Area	Height	Type	Width (50%)	Asym.	Resol.	Plates
2	No.		min	n.a.	%	mAU*min	mAU		min	EP	EP	EP
4	1		0,530	n.a.	7,96	16,3246	472,83	BMB*	0,033	1,02	1,77	1408
5	2		0,667	n.a.	92,04	188,8342	3073,22	BMB*	0,058	1,16	n.a.	737
6	Maximum			0,0000	92,04	188,8342	3073,22		0,058	1,16	1,77	1408
7	Minimum			0,0000	7,96	16,3246	472,83		0,033	1,02	1,77	737
8	Sum			0,0000	100,00	205,1588	3546,05					

4-Hydroxy thalidomide

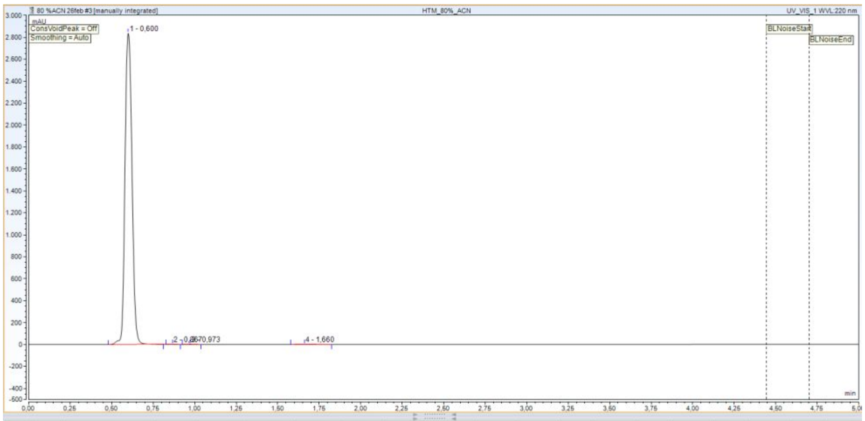

	A	B	C	D	E	F	G	H	I	J	K	L
1	Peak	Peak Name	Ret.Time	Amount	Rel.Area	Area	Height	Type	Width (50%)	Asym.	Resol.	Plates
2	No.		min	n.a.	%	mAU*min	mAU		min	EP	EP	EP
4	1		0,600	n.a.	99,83	138,3047	2837,23	BMB*	0,046	1,13	3,88	944
5	2		0,867	n.a.	0,03	0,0355	0,98	BMB*	0,035	1,04	1,40	3359
6	3		0,973	n.a.	0,06	0,0867	1,55	BMB*	0,055	1,11	5,42	1752
7	4		1,660	n.a.	0,09	0,1195	1,24	BMB*	0,095	1,18	n.a.	1696
8	Maximum			0,0000	99,83	138,3047	2837,23		0,095	1,18	5,42	3359
9	Minimum			0,0000	0,03	0,0355	0,98		0,035	1,04	1,40	944
10	Sum			0,0000	100,00	138,5465	2840,99					

Cis-OH-VH298 (S,S,S)

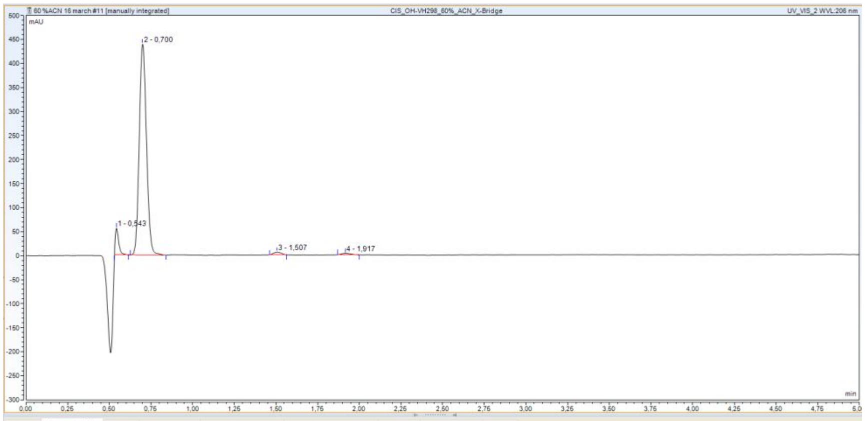

Detection | Component Table | Calibration | Peak Group Table | Chromatogram Subtraction | UV Spectral Library Screening | UV Settings | Advanced Settings | SST/IRC

Component Table

Group Area — Drag a column header here to group by that column. Run Component Table Wizard... Show Properties...

#	Name	Ret.Time	Window	Ref.UV.Spec.	Check Extr.	Match Crit.	Rel.Max.Dev.	Spec.Deriv.	Threshold	WL-Range	Channel	Eval.Type	Stand.Meth.	Cal.Type

	A	B	C	D	E	F	G	H	I	J	K	L
1	Peak	Peak Name	Ret.Time	Amount	Rel.Area	Area	Height	Type	Width (50%)	Asym.	Resol.	Plates
2	No.		min	n.a.	%	mAU*min	mAU		min	EP	EP	EP
4	1		0,543	n.a.	5,42	1,2900	55,41	MB*	0,021	2,04	2,72	3555
5	2		0,700	n.a.	92,52	22,0254	439,21	BMB*	0,047	1,12	9,72	1248
6	3		1,507	n.a.	1,23	0,2938	5,67	BMB*	0,051	1,13	4,11	4786

Summary | Peak Results | Peak Purity | Library Search | System Suitability Test | Calibration | Audit Trail

Cis-phenol-VH032 (S,S,S)

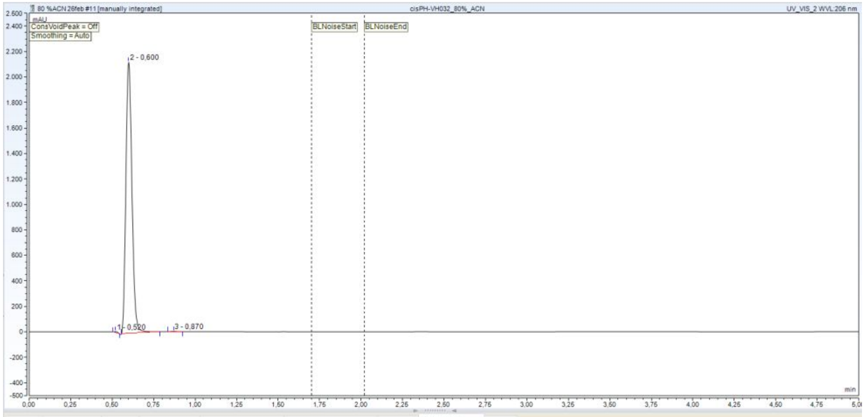

Peak No.	Peak Name	Ret.Time min	Amount n.a.	Rel.Area %	Area mAU*min	Height mAU	Type	Width (50%) min	Asym. EP	Resol. EP	Plates EP
1		0,520	n.a.	0,24	0,2173	7,27	BMB*	0,028	1,23	1,40	1909
2		0,600	n.a.	99,66	90,0895	2127,49	BMB*	0,039	1,17	4,20	1287
3		0,870	n.a.	0,10	0,0903	2,38	BMB*	0,036	1,10	n.a.	3161
Maximum			0,0000	99,66	90,0895	2127,49		0,039	1,23	4,20	3161
Minimum			0,0000	0,10	0,0903	2,38		0,028	1,10	1,40	1287
Sum			0,0000	100,00	90,3971	2137,14					

OH-VH298 (S,R,S)

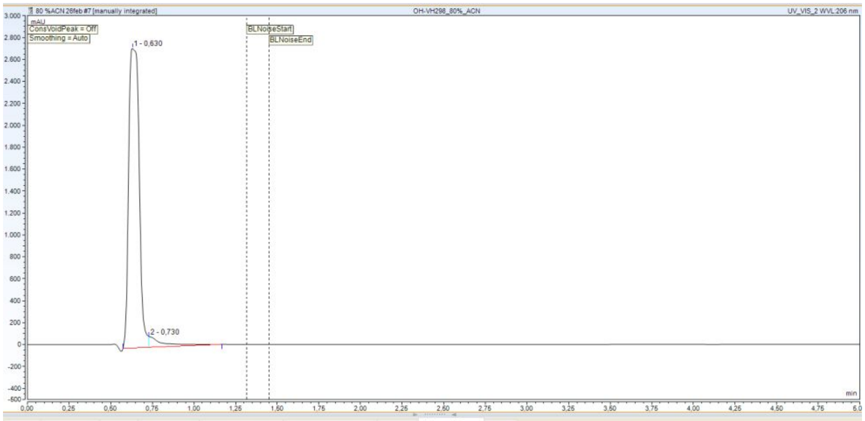

Peak No.	Peak Name	Ret.Time min	Amount n.a.	Rel.Area %	Area mAU*min	Height mAU	Type	Width (50%) min	Asym. EP	Resol. EP	Plates EP
1		0,630	n.a.	95,34	199,5436	2734,41	BM *	0,069	1,40	n.a.	456
2		0,730	n.a.	4,66	9,7425	98,72	MB*	n.a.	n.a.	n.a.	n.a.
Maximum			0,0000	95,34	199,5436	2734,41		0,069	1,40	0,00	456
Minimum			0,0000	4,66	9,7425	98,72		0,069	1,40	0,00	456
Sum			0,0000	100,00	209,2861	2833,13					

Phenol-VH032 (S,R,S)

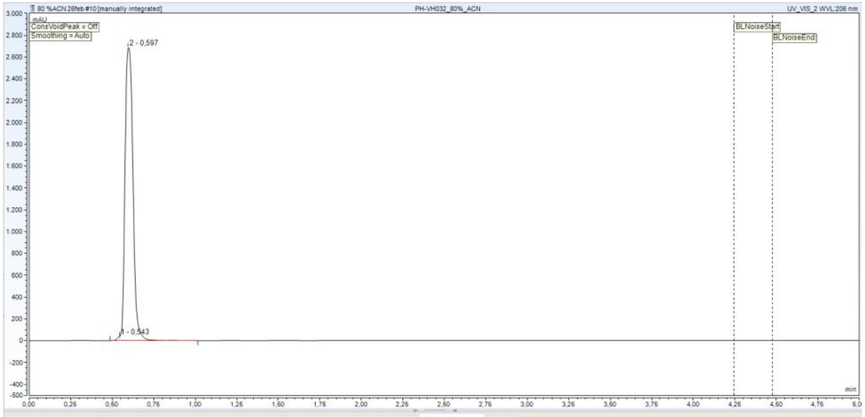

Peak No.	Peak Name	Ret.Time min	Amount n.a.	Rel.Area %	Area mAU*min	Height mAU	Type	Width (50%) min	Asym. EP	Resol. EP	Plates EP
1		0,543	n.a.	0,42	0,6213	39,10	BM *	n.a.	n.a.	n.a.	n.a.
2		0,597	n.a.	99,58	148,5364	2685,88	MB*	0,052	1,30	n.a.	728
Maximum			0,0000	99,58	148,5364	2685,88		0,052	1,30	0,00	728
Minimum			0,0000	0,42	0,6213	39,10		0,052	1,30	0,00	728
Sum			0,0000	100,00	149,1576	2724,98					

Pomalidomide

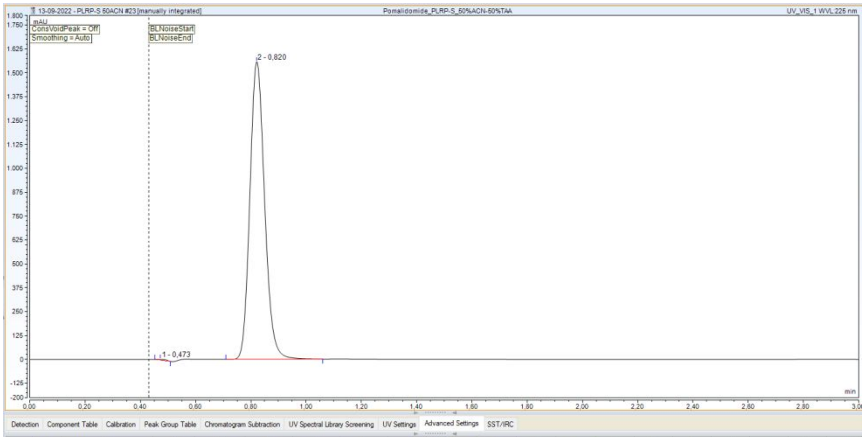

Peak No.	Peak Name	Ret.Time min	Amount n.a.	Rel.Area %	Area mAU*min	Height mAU	Type	Width (50%) min	Asym. EP	Resol. EP	Plates EP
1		0,473	n.a.	0,19	0,1798	3,93	BMB*	0,039	1,44	4,25	804
2		0,820	n.a.	99,81	96,2823	1557,18	BMB*	0,057	1,19	n.a.	1152
Maximum			0,0000	99,81	96,2823	1557,18		0,057	1,44	4,25	1152
Minimum			0,0000	0,19	0,1798	3,93		0,039	1,19	4,25	804
Sum			0,0000	100,00	96,4621	1561,11					

BI-0115

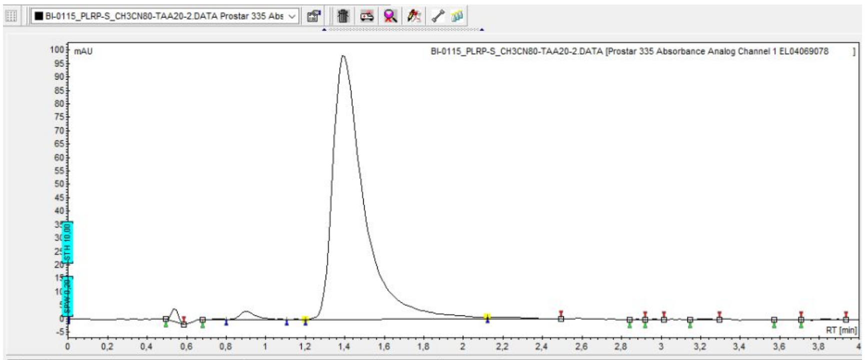

#	Name	Time [Min]	Quantity [% Area]	Height [mAU]	Area [mAU.Min]	Area % [%]	Lipophilicity inde
1	UNKNOWN	0,53	0,99	4,6	0,2	0,986	0,00
2	UNKNOWN	0,76	0,13	0,3	0,0	0,128	0,42
3	UNKNOWN	0,91	1,58	3,0	0,3	1,577	0,70
4	UNKNOWN	1,13	0,02	0,1	0,0	0,024	1,12
5	UNKNOWN	1,39	96,25	98,2	18,0	96,253	1,60
6	UNKNOWN	2,15	0,83	0,7	0,2	0,835	3,02
7	UNKNOWN	2,87	0,01	0,0	0,0	0,010	4,37
8	UNKNOWN	2,97	0,04	0,3	0,0	0,041	4,57
9	UNKNOWN	3,15	0,04	0,1	0,0	0,037	4,89
10	UNKNOWN	3,64	0,04	0,1	0,0	0,037	5,82
11	UNKNOWN	3,87	0,07	0,1	0,0	0,072	6,24
Total			100,00	107,6	18,7	100,000	32,75

BI-1580

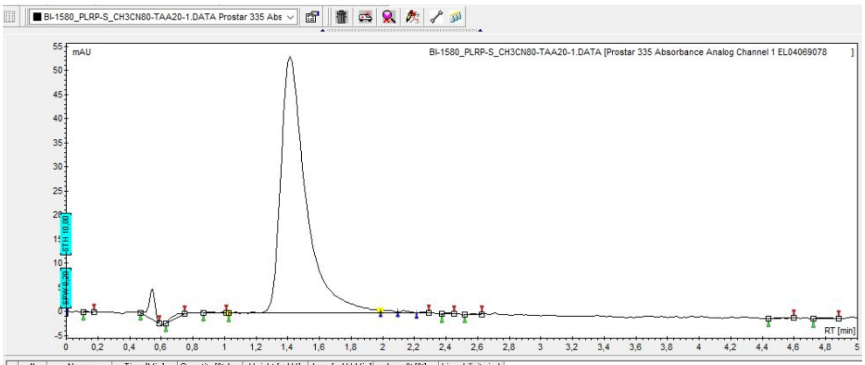

#	Name	Time [Min]	Quantity [% Area]	Height [mAU]	Area [mAU.Min]	Area % [%]	Lipophilicity inde
1	UNKNOWN	0,17	0,01	0,1	0,0	0,006	-0,68
2	UNKNOWN	0,55	2,72	6,3	0,3	2,717	0,02
3	UNKNOWN	0,69	0,51	0,7	0,1	0,514	0,30
4	UNKNOWN	0,92	0,12	0,1	0,0	0,116	0,72
5	UNKNOWN	1,41	95,40	53,2	9,8	95,397	1,65
6	UNKNOWN	2,01	0,51	0,6	0,1	0,507	2,77
7	UNKNOWN	2,15	0,35	0,4	0,0	0,351	3,02
8	UNKNOWN	2,25	0,09	0,2	0,0	0,091	3,22
9	UNKNOWN	2,41	0,06	0,2	0,0	0,059	3,52
10	UNKNOWN	2,57	0,06	0,2	0,0	0,058	3,82
11	UNKNOWN	4,51	0,06	0,1	0,0	0,055	7,44
12	UNKNOWN	4,77	0,13	0,2	0,0	0,130	7,94
Total			100,00	62,3	10,3	100,000	33,74

CPI203

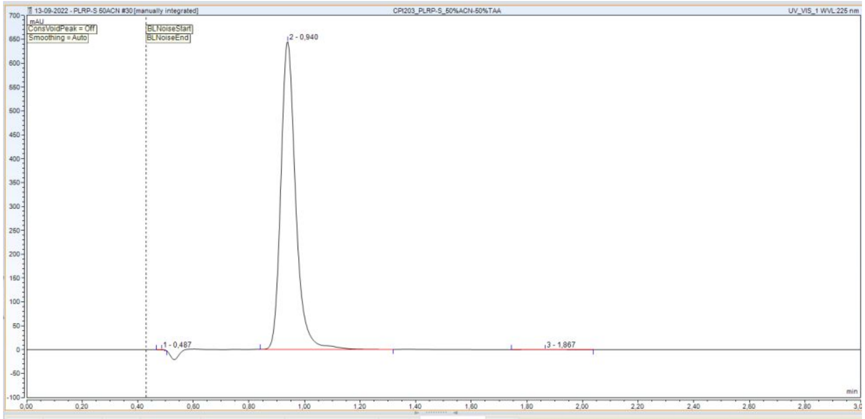

Peak No.	Peak Name	Ret.Time min	Amount n.a.	Rel.Area %	Area mAU*min	Height mAU	Type	Width (50%) min	Asym. EP	Resol. EP	Plates EP
1		0,487	n.a.	0,08	0,0309	1,22	BMB*	0,024	0,93	6,80	2206
2		0,940	n.a.	99,80	39,7274	644,16	BMB*	0,054	1,23	6,47	1661
3		1,867	n.a.	0,12	0,0472	0,37	BMB*	0,115	1,13	n.a.	1466
Maximum			0,0000	99,80	39,7274	644,16		0,115	1,23	6,80	2206
Minimum			0,0000	0,08	0,0309	0,37		0,024	0,93	6,47	1466
Sum			0,0000	100,00	39,8055	645,76					

HJB97

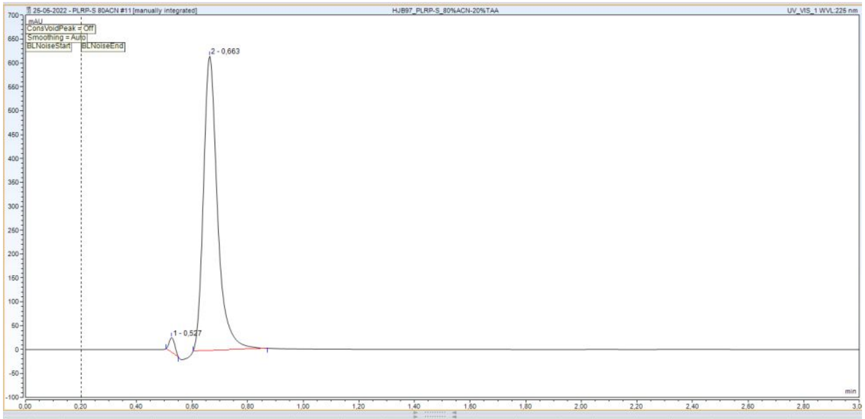

Peak No.	Peak Name	Ret.Time min	Amount n.a.	Rel.Area %	Area mAU*min	Height mAU	Type	Width (50%) min	Asym. EP	Resol. EP	Plates EP
1		0,527	n.a.	1,96	0,7182	31,12	BMB*	0,023	1,08	2,18	2917
2		0,663	n.a.	98,04	35,9848	616,02	BMB*	0,051	1,35	n.a.	939
Maximum			0,0000	98,04	35,9848	616,02		0,051	1,35	2,18	2917
Minimum			0,0000	1,96	0,7182	31,12		0,023	1,08	2,18	939
Sum			0,0000	100,00	36,7030	647,14					

MS-417

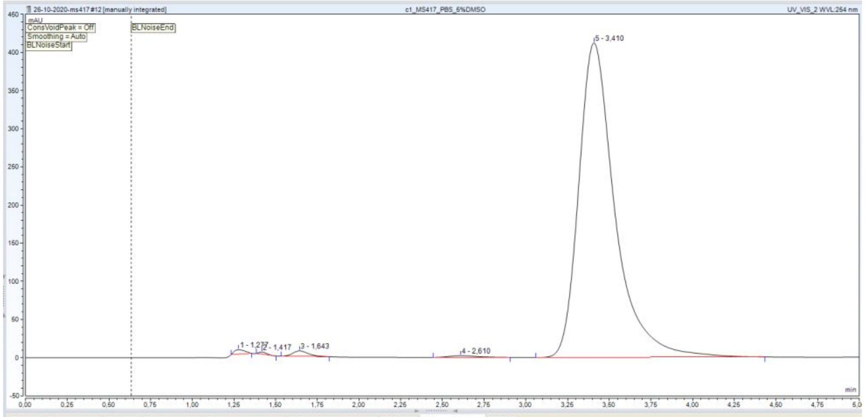

Peak No.	Peak Name	Ret.Time min	Amount n.a.	Rel.Area %	Area mAU*min	Height mAU	Type	Width (50%) min	Asym. EP	Resol. EP	Plates EP
1		1,277	n.a.	0,41	0,4263	5,86	BMB*	0,075	1,40	1,28	1612
2		1,417	n.a.	0,14	0,1443	2,62	BMB*	0,054	1,47	1,75	3778
3		1,643	n.a.	0,75	0,7943	7,25	BMB*	0,099	1,28	4,29	1531
4		2,610	n.a.	0,37	0,3945	2,13	BMB*	0,167	1,40	2,47	1354
5		3,410	n.a.	98,33	103,4864	412,04	BMB*	0,215	1,46	n.a.	1390
Maximum			0,0000	98,33	103,4864	412,04		0,215	1,47	4,29	3778
Minimum			0,0000	0,14	0,1443	2,13		0,054	1,28	1,28	1354
Sum			0,0000	100,00	105,2459	429,90					

OTX-015

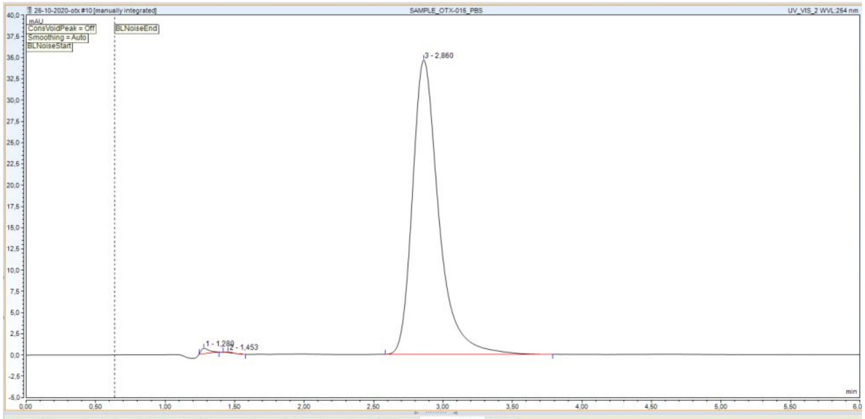

	A	B	C	D	E	F	G	H	I	J	K	L
1	Peak	Peak Name	Ret.Time	Amount	Rel.Area	Area	Height	Type	Width (50%)	Asym.	Resol.	Plates
2	No.		min	n.a.	%	mAU*min	mAU		min	EP	EP	EP
4	1		1,280	n.a.	0,56	0,0429	0,64	BMB*	0,062	1,88	1,75	2378
5	2		1,453	n.a.	0,06	0,0043	0,09	BMB*	0,055	1,28	6,76	3865
6	3		2,860	n.a.	99,38	7,5918	34,62	BMB*	0,190	1,40	n.a.	1250
7	Maximum			0,0000	99,38	7,5918	34,62		0,190	1,88	6,76	3865
8	Minimum			0,0000	0,06	0,0043	0,09		0,055	1,28	1,75	1250
9	Sum			0,0000	100,00	7,6390	35,35					

Cyclosporine

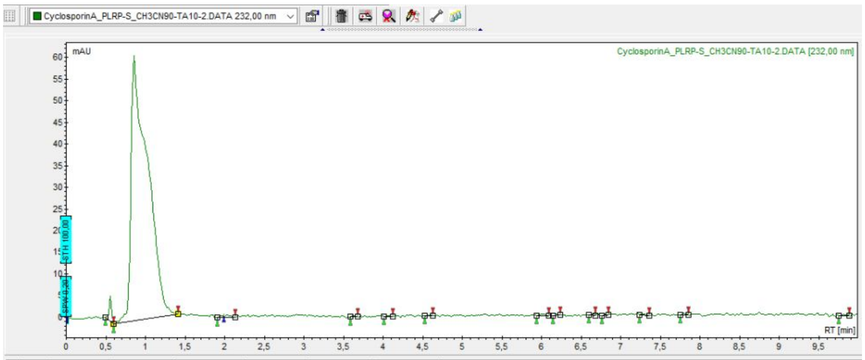

#	Name	Time [Min]	Quantity [% Area	Height [mAU]	Area [mAU.Min]	Area % [%]
1	UNKNOWN	0,56	1,26	6,0	0,2	1,261
2	UNKNOWN	0,85	97,64	61,2	14,9	97,639
3	UNKNOWN	1,96	0,09	0,3	0,0	0,093
4	UNKNOWN	2,03	0,23	0,5	0,0	0,232
5	UNKNOWN	3,63	0,07	0,2	0,0	0,073
6	UNKNOWN	4,07	0,09	0,3	0,0	0,094
7	UNKNOWN	4,59	0,05	0,2	0,0	0,051
8	UNKNOWN	6,03	0,16	0,4	0,0	0,161
9	UNKNOWN	6,17	0,05	0,2	0,0	0,047
10	UNKNOWN	6,65	0,07	0,2	0,0	0,071
11	UNKNOWN	6,80	0,00	0,2	0,0	0,002
12	UNKNOWN	7,27	0,08	0,2	0,0	0,084
13	UNKNOWN	7,79	0,08	0,2	0,0	0,079
14	UNKNOWN	9,80	0,11	0,2	0,0	0,113
Total			100,00	70,3	15,2	100,000

Everolimus

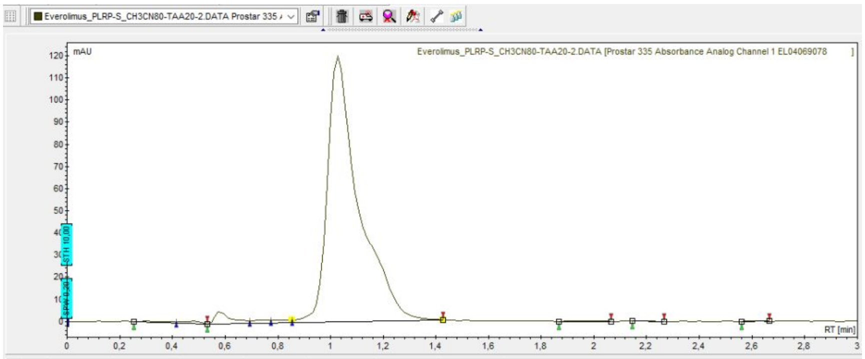

#	Name	Time [Min]	Quantity [% Area	Height [mAU]	Area [mAU.Min]	Area % [%]	Lipophilicity inde
1	UNKNOWN	0,41	0,29	0,6	0,1	0,295	-0,23
2	UNKNOWN	0,48	0,46	1,0	0,1	0,460	-0,10
3	UNKNOWN	0,57	2,11	5,2	0,4	2,110	0,07
4	UNKNOWN	0,73	0,59	1,4	0,1	0,589	0,37
5	UNKNOWN	0,77	0,52	1,2	0,1	0,521	0,45
6	UNKNOWN	1,03	95,87	120,0	16,5	95,870	0,92
7	UNKNOWN	1,93	0,10	0,2	0,0	0,099	2,62
8	UNKNOWN	2,23	0,03	0,1	0,0	0,035	3,17
9	UNKNOWN	2,63	0,02	0,1	0,0	0,021	3,92
Total			100,00	129,7	17,2	100,000	11,20

Pimecrolimus

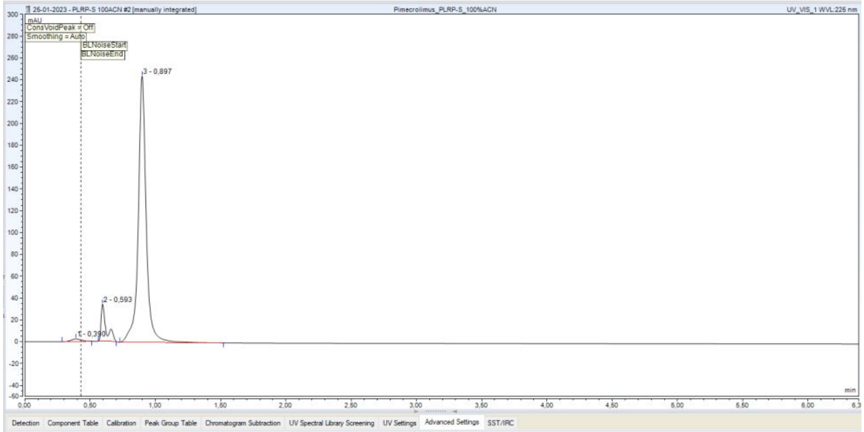

Peak No.	Peak Name	Ret.Time min	Amount n.a.	Rel.Area %	Area mAU*min	Height mAU	Type	Width (50%) min	Asym. EP	Resol. EP	Plates EP
1		0,390	n.a.	1,12	0,2136	2,47	BMB*	0,078	1,19	2,21	138
2		0,593	n.a.	8,00	1,5247	33,86	BMB*	0,030	2,72	4,10	2111
3		0,897	n.a.	90,88	17,3233	244,05	BMB*	0,057	0,99	n.a.	1372
Maximum			0,0000	90,88	17,3233	244,05		0,078	2,72	4,10	2111
Minimum			0,0000	1,12	0,2136	2,47		0,030	0,99	2,21	138
Sum			0,0000	100,00	19,0617	280,38					

Sirolimus

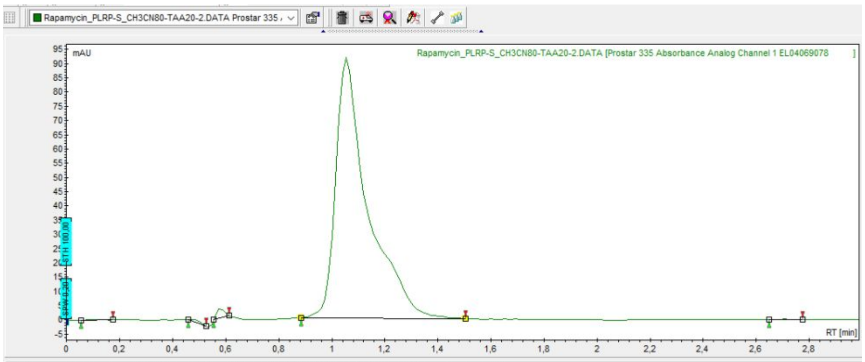

#	Name	Time [Min]	Quantity [% Area]	Height [mAU]	Area [mAU.Min]	Area % [%]	Lipophilicity inde
1	UNKNOWN	0,11	0,11	0,2	0,0	0,113	-0,80
4	UNKNOWN	0,49	0,23	1,0	0,0	0,229	-0,08
3	UNKNOWN	0,57	0,83	3,2	0,1	0,828	0,07
2	UNKNOWN	1,05	98,77	91,4	12,9	98,769	0,97
5	UNKNOWN	2,71	0,06	0,2	0,0	0,061	4,07
Total			100,00	96,1	13,0	100,000	4,24

Temsirolimus

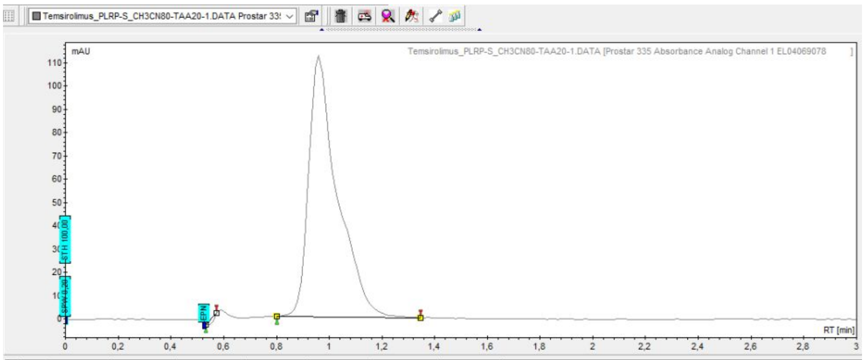

#	Name	Time [Min]	Quantity [% Area]	Height [mAU]	Area [mAU.Min]	Area % [%]	Lipophilicity inde
1	UNKNOWN	0,56	0,30	1,7	0,0	0,304	0,05
2	UNKNOWN	0,96	99,70	111,9	14,1	99,696	0,80
Total			100,00	113,6	14,1	100,000	0,85

Atazanavir

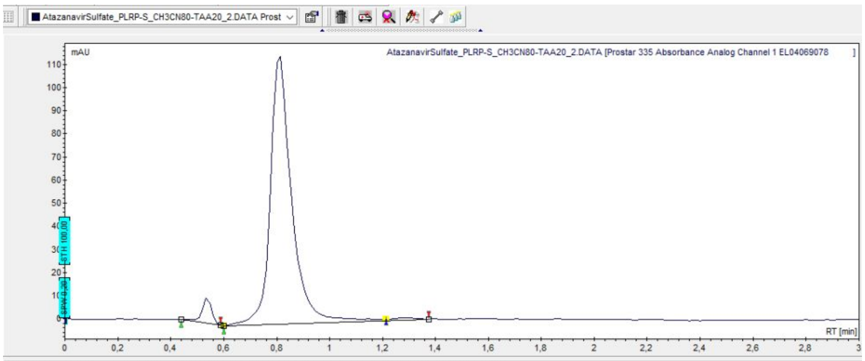

#	Name	Time [Min]	Quantity [% Area	Height [mAU]	Area [mAU.Min]	Area % [%]	Lipophilicity inde
1	UNKNOWN	0,53	4,08	10,9	0,5	4,079	0,00
2	UNKNOWN	0,81	95,22	115,5	10,9	95,223	0,52
3	UNKNOWN	1,28	0,70	0,9	0,1	0,698	1,40
Total			100,00	127,2	11,4	100,000	1,92

Nelfinavir

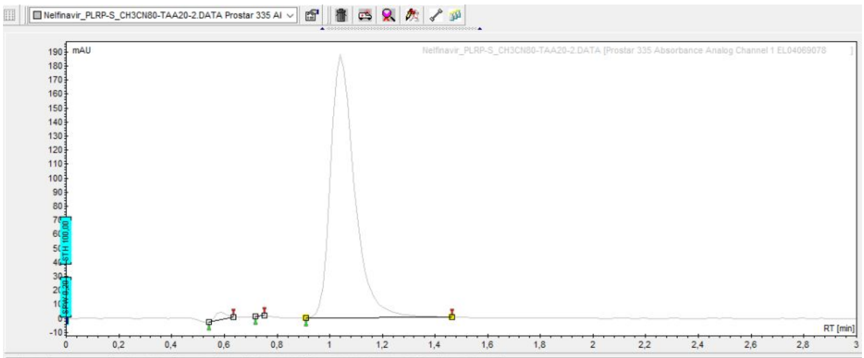

#	Name	Time [Min]	Quantity [% Area	Height [mAU]	Area [mAU.Min]	Area % [%]	Lipophilicity inde
3	UNKNOWN	0,59	1,22	5,3	0,2	1,223	0,10
2	UNKNOWN	0,73	0,04	0,4	0,0	0,041	0,37
1	UNKNOWN	1,04	98,74	187,4	19,8	98,737	0,95
Total			100,00	193,1	20,0	100,000	1,42

Paclitaxel

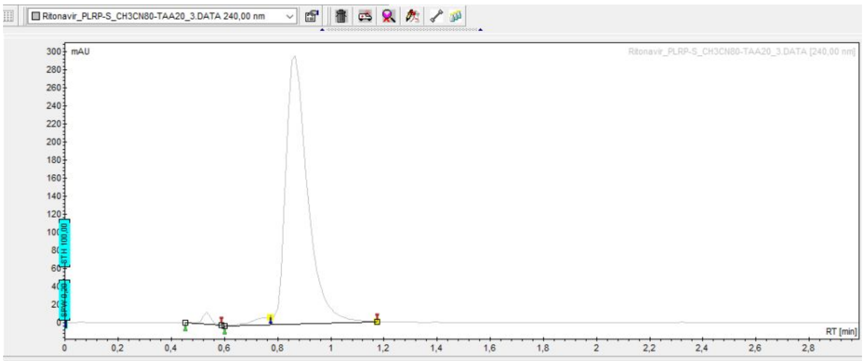

#	Name	Time [Min]	Quantity [% Area	Height [mAU]	Area [mAU.Min]	Area % [%]
1	UNKNOWN	0,53	1,75	13,6	0,5	1,754
2	UNKNOWN	0,75	1,94	7,2	0,6	1,942
3	UNKNOWN	0,87	96,30	296,4	28,1	96,304
Total			100,00	317,2	29,2	100,000

Ritonavir

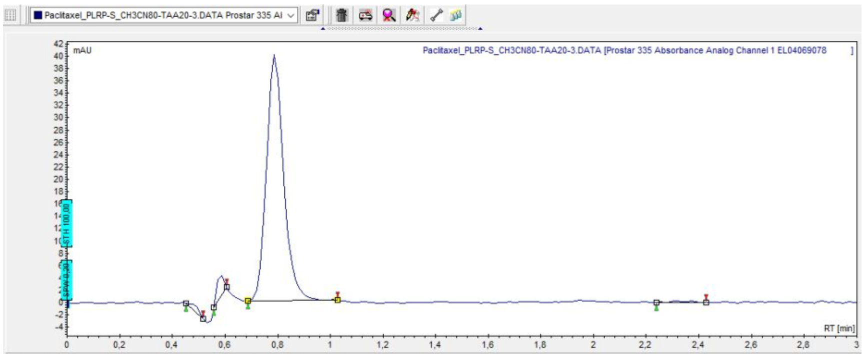

#	Name	Time [Min]	Quantity [% Area	Height [mAU]	Area [mAU.Min]	Area % [%]	Lipophilicity inde
3	UNKNOWN	0,49	1,10	1,0	0,0	1,103	-0,08
4	UNKNOWN	0,57	3,16	3,4	0,1	3,164	0,07
2	UNKNOWN	0,79	95,33	39,9	3,0	95,327	0,47
1	UNKNOWN	2,35	0,41	0,1	0,0	0,406	3,39
Total			100,00	44,3	3,2	100,000	3,87

Saquinavir

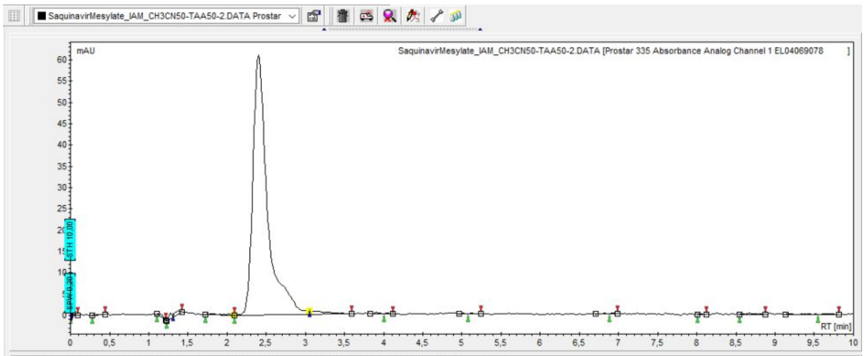

#	Name	Time [Min]	Quantity [% Area	Height [mAU]	Area [mAU.Min]	Area % [%]	Lipophilicity inde
1	UNKNOWN	0,05	0,07	0,2	0,0	0,071	-0,90
2	UNKNOWN	0,36	0,04	0,0	0,0	0,037	-0,33
3	UNKNOWN	1,16	0,33	0,7	0,0	0,326	1,17
4	UNKNOWN	1,27	0,47	1,3	0,1	0,466	1,37
5	UNKNOWN	1,36	0,47	0,7	0,1	0,472	1,55
6	UNKNOWN	2,01	0,29	0,3	0,0	0,295	2,77
7	UNKNOWN	2,40	96,28	60,9	13,2	96,285	3,49
8	UNKNOWN	3,11	1,26	0,6	0,2	1,261	4,82
9	UNKNOWN	4,05	0,09	0,3	0,0	0,095	6,59
10	UNKNOWN	5,12	0,10	0,2	0,0	0,103	8,59
11	UNKNOWN	6,93	0,09	0,2	0,0	0,091	11,98
12	UNKNOWN	8,05	0,05	0,1	0,0	0,051	14,08
13	UNKNOWN	8,68	0,22	0,3	0,0	0,224	15,25
14	UNKNOWN	9,68	0,23	0,1	0,0	0,226	17,13
Total			100,00	66,0	13,7	100,000	87,57

Telaprevir

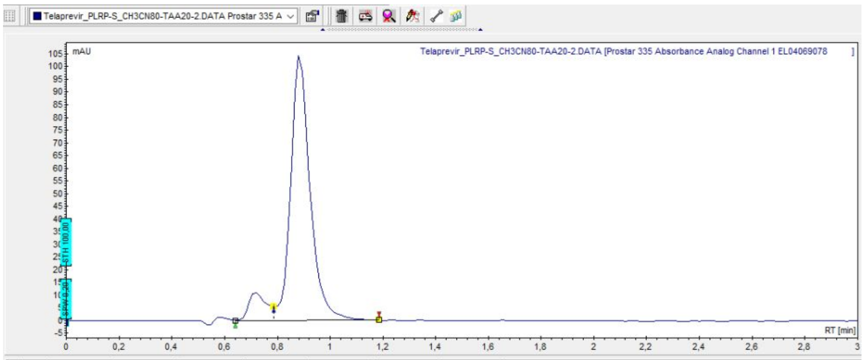

#	Name	Time [Min]	Quantity [% Area	Height [mAU]	Area [mAU.Min]	Area % [%]	Lipophilicity inde
1	UNKNOWN	0,72	8,81	11,0	0,9	8,810	0,35
2	UNKNOWN	0,88	91,19	104,1	8,8	91,190	0,65
Total			100,00	115,0	9,7	100,000	1,00

PEG_4-PH-NH_2-Pomalidomide

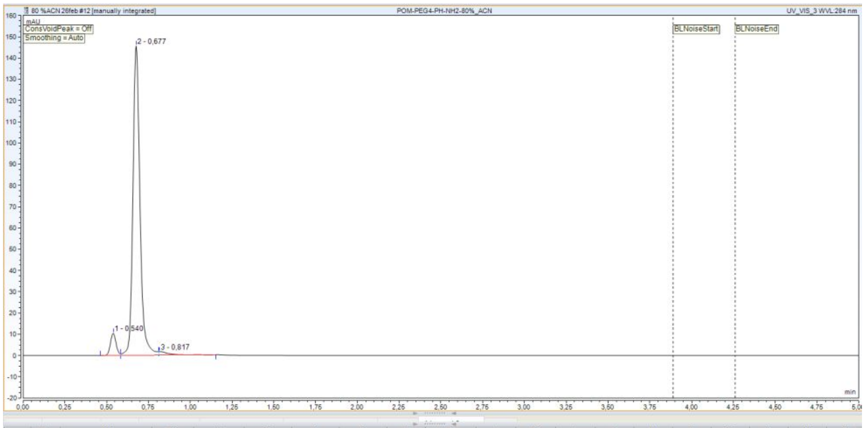

	A	B	C	D	E	F	G	H	I	J	K	L
1	Peak	Peak Name	Ret.Time	Amount	Rel.Area	Area	Height	Type	Width (50%)	Asym.	Resol.	Plates
2	No.		min	n.a.	%	mAU*min	mAU		min	EP	EP	EP
4	1		0,540	n.a.	5,33	0,4068	10,51	BM *	0,036	n.a.	2,04	1245
5	2		0,677	n.a.	93,23	7,1174	145,59	M *	0,043	1,20	n.a.	1379
6	3		0,817	n.a.	1,44	0,1097	1,61	MB*	n.a.	n.a.	n.a.	n.a.
7	Maximum			0,0000	93,23	7,1174	145,59		0,043	1,20	2,04	1379
8	Minimum			0,0000	1,44	0,1097	1,61		0,036	1,20	2,04	1245
9	Sum			0,0000	100,00	7,6339	157,71					

ARV-825

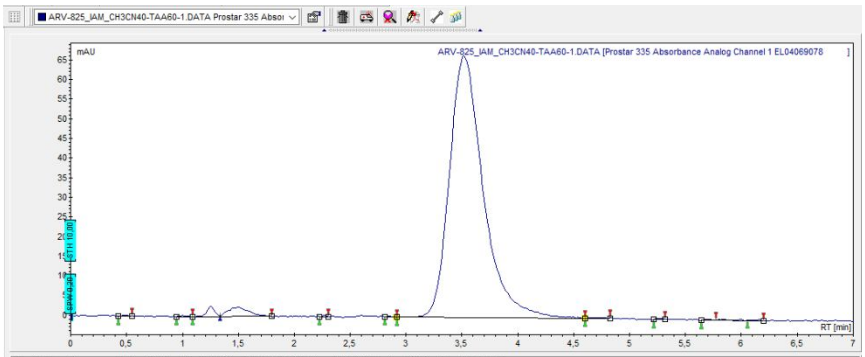

#	Name	Time [Min]	Quantity [% Area]	Height [mAU]	Area [mAU.Min]	Area % [%]	Lipophilicity inde
1	UNKNOWN	0,48	0,06	0,2	0,0	0,058	-0,10
2	UNKNOWN	0,99	0,08	0,3	0,0	0,083	0,85
3	UNKNOWN	1,25	0,90	2,6	0,2	0,896	1,35
4	UNKNOWN	1,51	2,11	2,4	0,5	2,111	1,82
5	UNKNOWN	2,28	0,05	0,3	0,0	0,052	3,27
6	UNKNOWN	2,84	0,02	0,1	0,0	0,024	4,32
7	UNKNOWN	3,52	96,45	66,5	23,3	96,449	5,59
8	UNKNOWN	4,63	0,09	0,2	0,0	0,087	7,66
9	UNKNOWN	5,25	0,06	0,3	0,0	0,056	8,84
10	UNKNOWN	5,72	0,10	0,3	0,0	0,095	9,71
11	UNKNOWN	6,16	0,09	0,3	0,0	0,089	10,54
Total			100,00	73,2	24,2	100,000	53,84

BI-0319

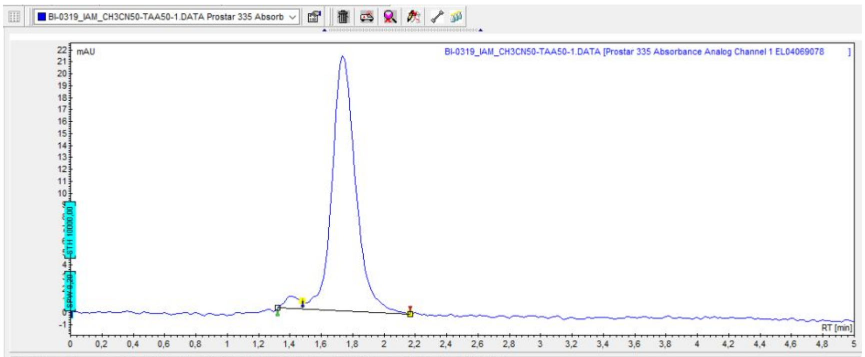

#	Name	Time [Min]	Quantity [% Area]	Height [mAU]	Area [mAU.Min]	Area % [%]	Lipophilicity inde
1	UNKNOWN	1,41	2,85	1,0	0,1	2,847	1,65
2	UNKNOWN	1,73	97,15	21,3	3,7	97,153	2,25
Total			100,00	22,3	3,8	100,000	3,89

BI-3663

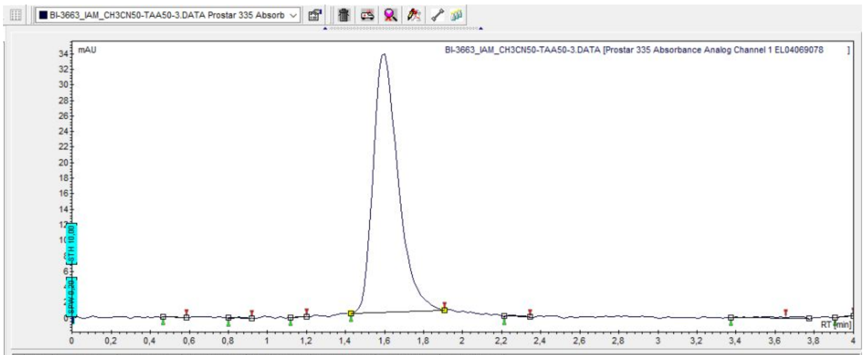

#	Name	Time [Min]	Quantity [% Area	Height [mAU]	Area [mAU.Min]	Area % [%]	Lipophilicity inde
1	UNKNOWN	0,59	0,04	0,0	0,0	0,042	0,10
2	UNKNOWN	0,85	0,15	0,2	0,0	0,150	0,60
3	UNKNOWN	1,17	0,08	0,1	0,0	0,078	1,20
4	UNKNOWN	1,60	98,87	33,2	4,9	98,875	2,00
5	UNKNOWN	2,25	0,21	0,2	0,0	0,215	3,22
6	UNKNOWN	3,55	0,51	0,1	0,0	0,514	5,64
7	UNKNOWN	3,96	0,13	0,1	0,0	0,126	6,42
Total			100,00	33,9	5,0	100,000	19,17

BI-4206

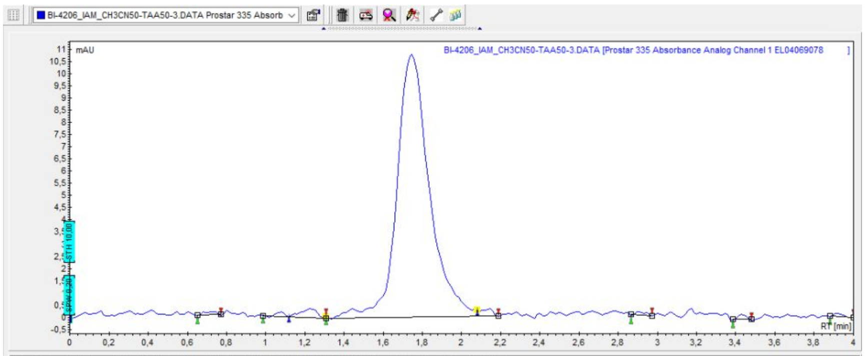

#	Name	Time [Min]	Quantity [% Area	Height [mAU]	Area [mAU.Min]	Area % [%]	Lipophilicity inde
1	UNKNOWN	0,69	0,37	0,1	0,0	0,374	0,30
2	UNKNOWN	1,08	0,44	0,2	0,0	0,440	1,02
3	UNKNOWN	1,23	1,64	0,4	0,0	1,643	1,30
4	UNKNOWN	1,75	95,55	10,8	2,1	95,549	2,27
5	UNKNOWN	2,15	1,02	0,3	0,0	1,017	3,02
6	UNKNOWN	2,91	0,32	0,1	0,0	0,324	4,44
7	UNKNOWN	3,43	0,41	0,2	0,0	0,406	5,42
8	UNKNOWN	3,91	0,25	0,1	0,0	0,246	6,32
Total			100,00	12,2	2,2	100,000	24,08

BRD4 degrader AT1

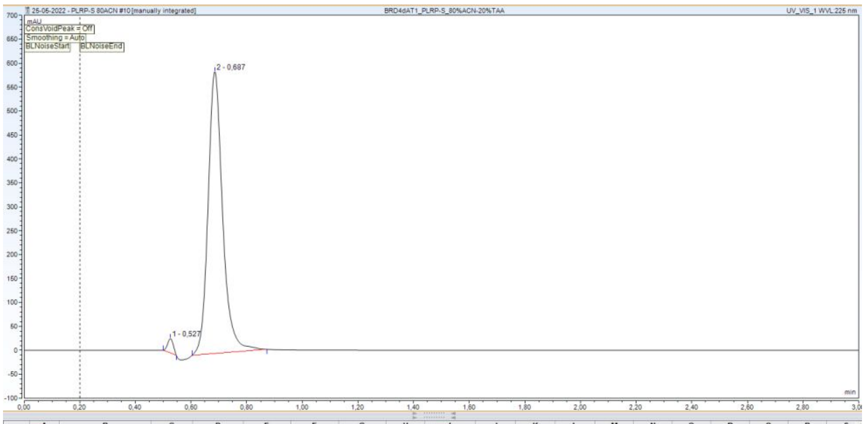

	A	B	C	D	E	F	G	H	I	J	K	L
1	Peak	Peak Name	Ret.Time	Amount	Rel.Area	Area	Height	Type	Width (50%)	Asym.	Resol.	Plates
2	No.		min	n.a.	%	mAU*min	mAU		min	EP	EP	EP
4	1		0,527	n.a.	1,95	0,7088	30,17	BMB*	0,023	0,91	2,48	2849
5	2		0,687	n.a.	98,05	35,6480	588,24	BMB*	0,053	1,17	n.a.	928
6	Maximum			0,0000	98,05	35,6480	588,24		0,053	1,17	2,48	2849
7	Minimum			0,0000	1,95	0,7088	30,17		0,023	0,91	2,48	928
8	Sum			0,0000	100,00	36,3568	618,40					

Cis-MZ1

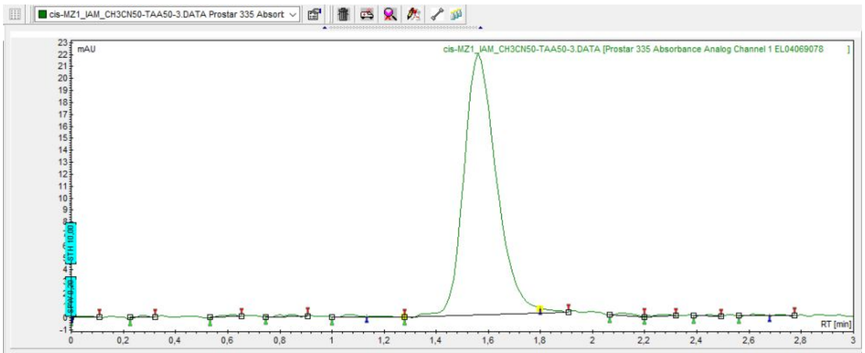

#	Name	Time [Min]	Quantity [% Area]	Height [mAU]	Area [mAU.Min]	Area % [%]	Lipophilicity index
1	UNKNOWN	0,05	0,18	0,0	0,0	0,177	-0,90
2	UNKNOWN	0,28	0,12	0,1	0,0	0,117	-0,48
3	UNKNOWN	0,60	0,25	0,1	0,0	0,247	0,12
4	UNKNOWN	0,84	0,30	0,2	0,0	0,300	0,57
5	UNKNOWN	1,07	0,32	0,2	0,0	0,317	1,00
6	UNKNOWN	1,19	0,27	0,1	0,0	0,267	1,22
7	UNKNOWN	1,56	96,91	21,9	3,2	96,905	1,92
8	UNKNOWN	1,83	0,61	0,3	0,0	0,608	2,42
9	UNKNOWN	2,13	0,43	0,2	0,0	0,428	3,00
10	UNKNOWN	2,25	0,27	0,1	0,0	0,272	3,22
11	UNKNOWN	2,49	0,10	0,1	0,0	0,096	3,67
12	UNKNOWN	2,63	0,17	0,1	0,0	0,166	3,92
13	UNKNOWN	2,72	0,10	0,1	0,0	0,099	4,09
Total			100,00	23,5	3,3	100,000	23,78

CRBN-6-5-5-VHL

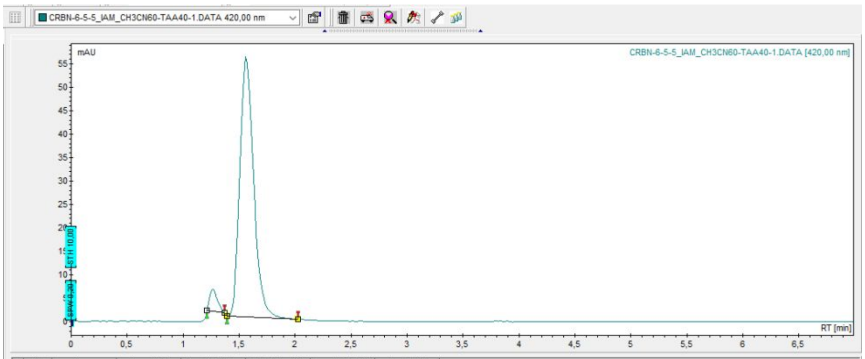

#	Name	Time [Min]	Quantity [% Area]	Height [mAU]	Area [mAU.Min]	Area % [%]
2	UNKNOWN	1,27	4,63	4,7	0,4	4,626
1	UNKNOWN	1,56	95,37	55,3	7,9	95,374
Total			100,00	60,0	8,3	100,000

dBET1

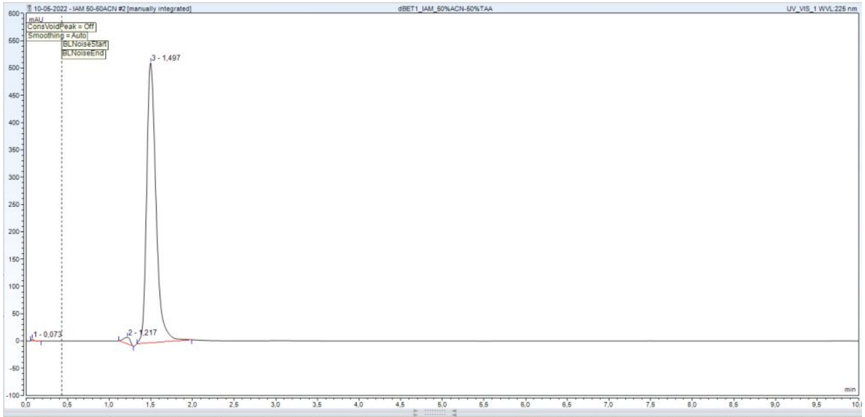

	A	B	C	D	E	F	G	H	I	J	K	L
1	Peak	Peak Name	Ret.Time	Amount	Rel.Area	Area	Height	Type	Width (50%)	Asym.	Resol.	Plates
2	No.		min	n.a.	%	mAU*min	mAU		min	EP	EP	EP
4	1		0,073	n.a.	0,10	0,0685	2,99	BMB*	0,025	1,50	11,61	48
5	2		1,217	n.a.	1,61	1,0921	11,85	BMB*	0,091	0,85	1,59	982
6	3		1,497	n.a.	98,29	66,8026	512,95	BMB*	0,116	1,38	n.a.	919
7	Maximum			0,0000	98,29	66,8026	512,95		0,116	1,50	11,61	982
8	Minimum			0,0000	0,10	0,0685	2,99		0,025	0,85	1,59	48
9	Sum			0,0000	100,00	67,9632	527,79					

dBET57

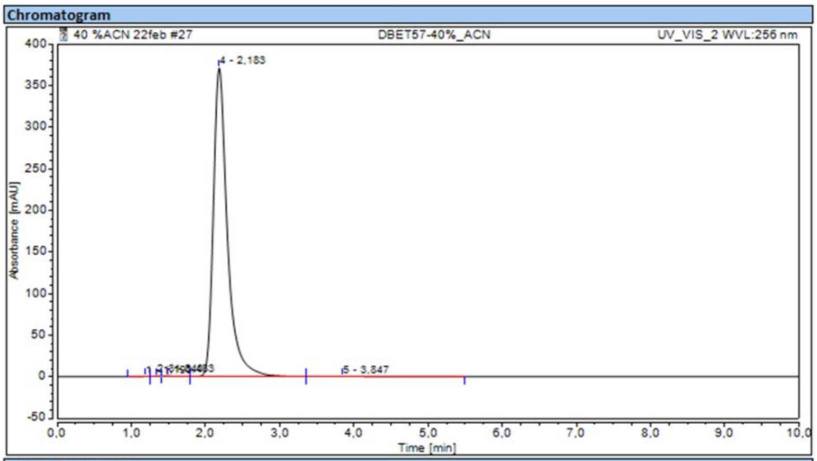

Integration Results

No.	Peak Name	Retention Time min	Area mAU*min	Height mAU	Relative Area %	Relative Height %	Amount n.a.
1		1,190	0,045	0,497	0,06	0,13	n.a.
2		1.340	0.113	0.925	0.14	0.25	n.a.
3		1,483	0,221	0,965	0,27	0,26	n.a.
4		2.183	81.690	371.414	99.05	99.24	n.a.
5		3.847	0.408	0.442	0.49	0.12	n.a.
**Total:**			**82,477**	**374,243**	**100,00**	**100,00**	

dBET6

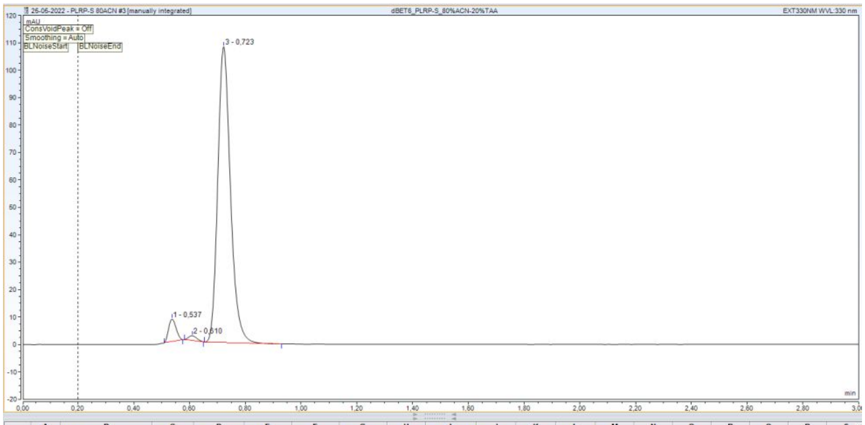

	A	B	C	D	E	F	G	H	I	J	K	L
1	Peak	Peak Name	Ret.Time	Amount	Rel.Area	Area	Height	Type	Width (50%)	Asym.	Resol.	Plates
2	No.		min	n.a.	%	mAU*min	mAU		min	EP	EP	EP
4	1		0,537	n.a.	4,25	0,2549	8,19	BMB*	0,030	1,21	1,35	1745
5	2		0,610	n.a.	0,96	0,0576	1,68	BMB*	0,034	1,20	1,64	1795
6	3		0,723	n.a.	94,79	5,6852	107,88	BMB*	0,048	1,20	n.a.	1272
7	Maximum			0,0000	94,79	5,6852	107,88		0,048	1,21	1,64	1795
8	Minimum			0,0000	0,96	0,0576	1,68		0,030	1,20	1,35	1272
9	Sum			0,0000	100,00	5,9977	117,75					

Gefitinib-based PROTAC 3

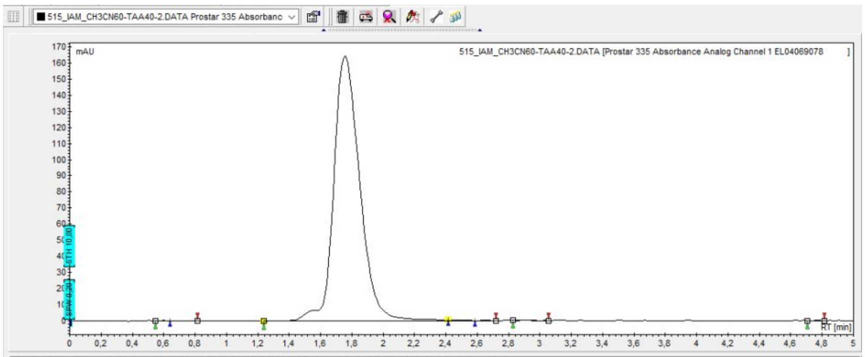

#	Name	Time [Min]	Quantity [% Area	Height [mAU]	Area [mAU.Min]	Area % [%]	Lipophilicity inde
1	UNKNOWN	0,60	0,03	0,3	0,0	0,030	0,12
2	UNKNOWN	0,72	0,04	0,1	0,0	0,042	0,35
3	UNKNOWN	1,76	99,63	164,3	31,6	99,632	2,30
4	UNKNOWN	2,47	0,17	0,5	0,1	0,172	3,62
5	UNKNOWN	2,65	0,04	0,1	0,0	0,042	3,97
6	UNKNOWN	2,91	0,05	0,1	0,0	0,050	4,44
7	UNKNOWN	4,77	0,03	0,3	0,0	0,032	7,94
Total			100,00	165,7	31,8	100,000	22,74

MZ1

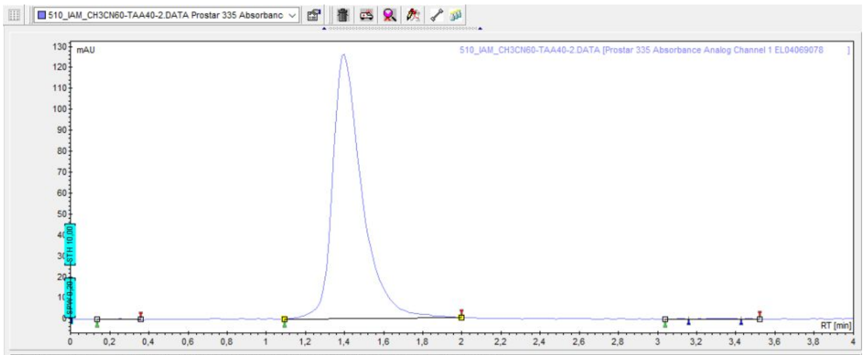

#	Name	Time [Min]	Quantity [% Area]	Height [mAU]	Area [mAU.Min]	Area % [%]	Lipophilicity inde
1	UNKNOWN	0,25	0,09	0,3	0,0	0,093	-0,53
2	UNKNOWN	1,40	99,61	126,0	22,0	99,607	1,62
3	UNKNOWN	3,11	0,03	0,1	0,0	0,032	4,82
4	UNKNOWN	3,35	0,22	0,3	0,0	0,224	5,27
5	UNKNOWN	3,47	0,04	0,3	0,0	0,044	5,49
Total			100,00	127,0	22,0	100,000	16,67

MZP-54

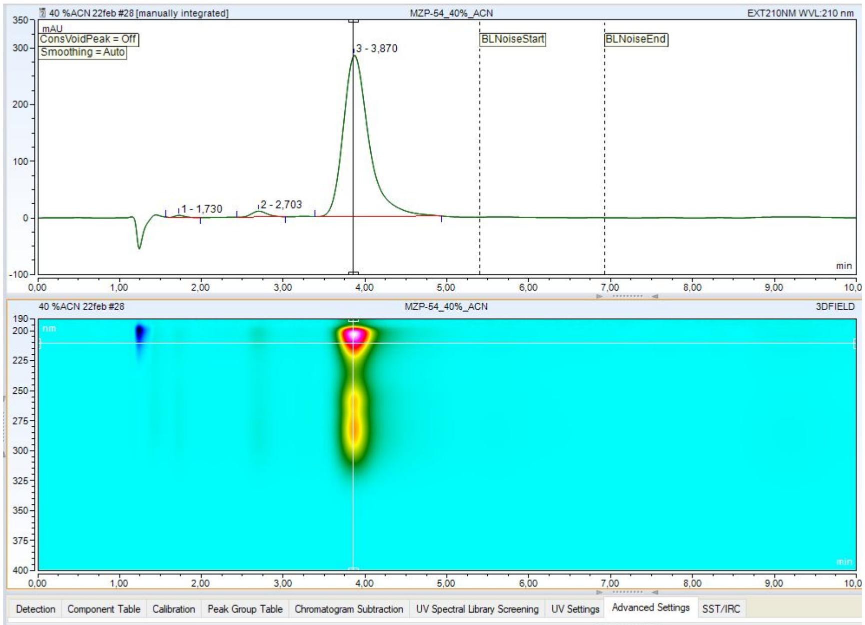

	A	B	C	D	E	F	G	H	I	J	K	L
1	Peak	Peak Name	Ret.Time	Amount	Rel.Area	Area	Height	Type	Width (50%)	Asym.	Resol.	Plates
2	No.		min	n.a.	%	mAU*min	mAU		min	EP	EP	EP
4	1		1,730	n.a.	0,40	0,4294	3,54	BMB*	0,119	1,39	3,53	1165
5	2		2,703	n.a.	2,15	2,2974	10,36	BMB*	0,206	1,20	2,64	951
6	3		3,870	n.a.	97,45	104,0248	285,21	BMB*	0,315	1,42	n.a.	834
7	Maximum			0,0000	97,45	104,0248	285,21		0,315	1,42	3,53	1165
8	Minimum			0,0000	0,40	0,4294	3,54		0,119	1,20	2,64	834
9	Sum			0,0000	100,00	106,7516	299,10					

PROTAC BET Degrader-10

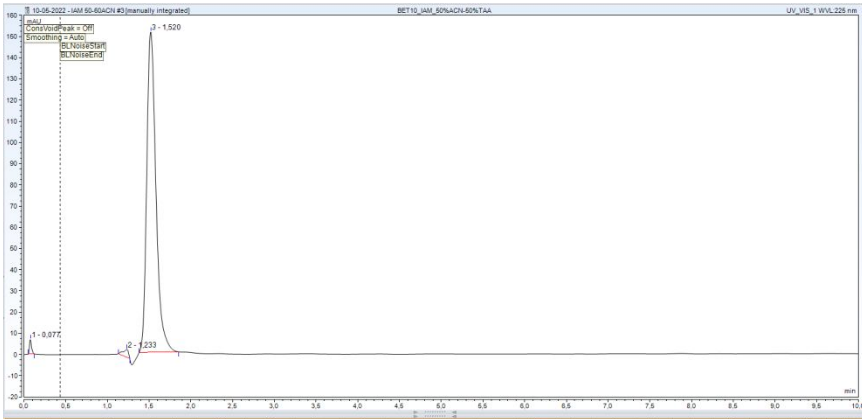

	A	B	C	D	E	F	G	H	I	J	K	L
1	Peak	Peak Name	Ret.Time	Amount	Rel.Area	Area	Height	Type	Width (50%)	Asym.	Resol.	Plates
2	No.		min	n.a.	%	mAU*min	mAU		min	EP	EP	EP
4	1		0,077	n.a.	0,96	0,1902	6,73	BMB*	0,026	1,38	14,52	47
5	2		1,233	n.a.	1,37	0,2716	3,72	BMB*	0,068	0,66	1,85	1835
6	3		1,520	n.a.	97,66	19,2909	150,98	BMB*	0,115	1,35	n.a.	962
7	Maximum			0,0000	97,66	19,2909	150,98		0,115	1,38	14,52	1835
8	Minimum			0,0000	0,96	0,1902	3,72		0,026	0,66	1,85	47
9	Sum			0,0000	100,00	19,7527	161,43					

PROTAC FAK-degrader 1

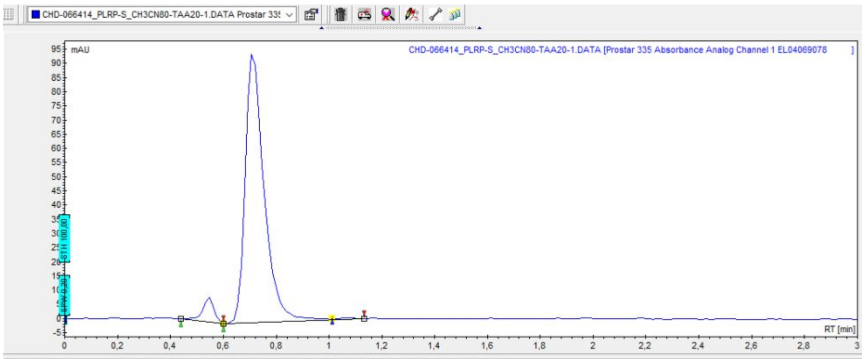

#	Name	Time [Min]	Quantity [% Area]	Height [mAU]	Area [mAU.Min]	Area % [%]	Lipophilicity inde
1	UNKNOWN	0,55	4,99	8,5	0,4	4,991	0,02
2	UNKNOWN	0,71	94,57	94,5	7,9	94,568	0,32
3	UNKNOWN	1,05	0,44	0,4	0,0	0,442	0,97
Total			100,00	103,5	8,3	100,000	1,32

PROTAC Mcl degrader-1

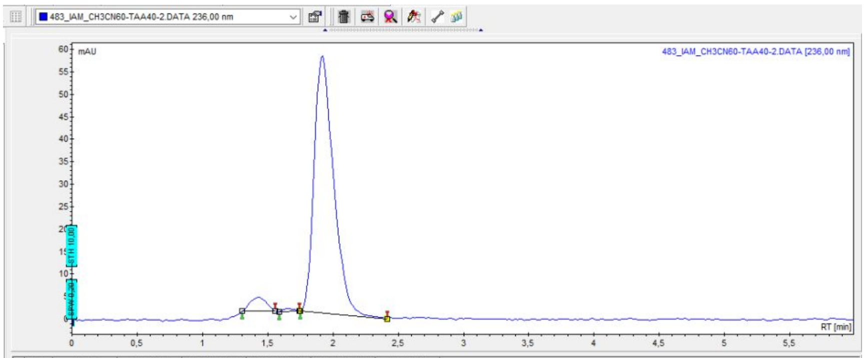

#	Name	Time [Min]	Quantity [% Area]	Height [mAU]	Area [mAU.Min]	Area % [%]
3	UNKNOWN	1,43	4,27	3,1	0,4	4,272
2	UNKNOWN	1,65	0,56	0,6	0,1	0,564
1	UNKNOWN	1,92	95,16	57,1	9,9	95,163
Total			100,00	60,9	10,4	100,000

PROTAC-1

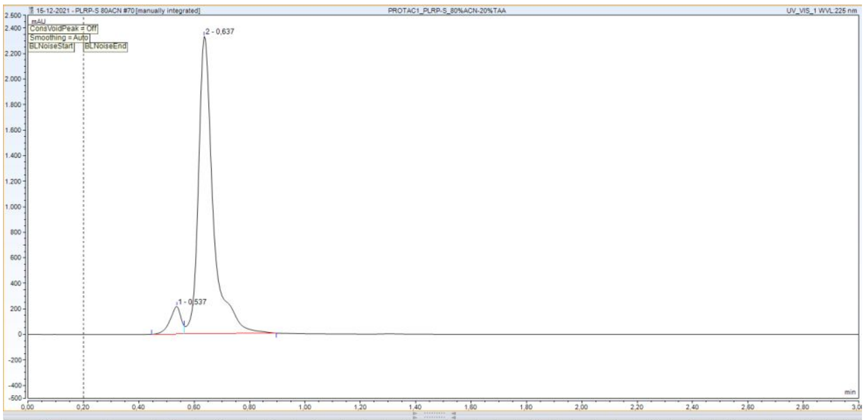

	A	B	C	D	E	F	G	H	I	J	K	L
1	Peak	Peak Name	Ret.Time	Amount	Rel.Area	Area	Height	Type	Width (50%)	Asym.	Resol.	Plates
2	No.		min	n.a.	%	mAU*min	mAU		min	EP	EP	EP
4	1		0,537	n.a.	6,38	9,8332	216,21	BM *	0,043	n.a.	1,29	875
5	2		0,637	n.a.	93,62	144,2174	2330,82	MB*	0,049	1,65	n.a.	934
6	Maximum			0,0000	93,62	144,2174	2330,82		0,049	1,65	1,29	934
7	Minimum			0,0000	6,38	9,8332	216,21		0,043	1,65	1,29	875
8	Sum			0,0000	100,00	154,0506	2547,03					

ZXH-3-26

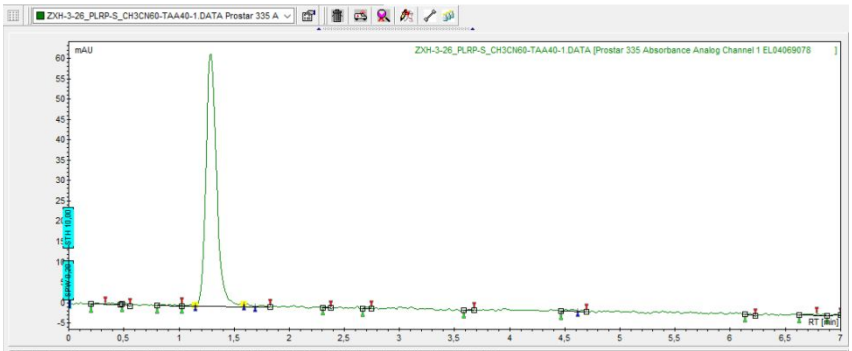

#	Name	Time [Min]	Quantity [% Area	Height [mAU]	Area [mAU.Min]	Area % [%]	Lipophilicity inde
1	UNKNOWN	0,28	0,21	0,2	0,0	0,212	-0,48
15	UNKNOWN	0,52	0,23	0,5	0,0	0,230	-0,03
2	UNKNOWN	0,95	0,37	0,1	0,0	0,373	0,77
3	UNKNOWN	1,15	0,27	0,3	0,0	0,275	1,15
4	UNKNOWN	1,28	96,31	61,7	6,9	96,313	1,40
5	UNKNOWN	1,61	0,60	0,7	0,0	0,598	2,02
6	UNKNOWN	1,75	0,39	0,4	0,0	0,385	2,27
7	UNKNOWN	2,37	0,09	0,1	0,0	0,095	3,44
8	UNKNOWN	2,71	0,22	0,4	0,0	0,223	4,07
9	UNKNOWN	3,64	0,03	0,2	0,0	0,034	5,82
10	UNKNOWN	4,53	0,47	0,1	0,0	0,472	7,49
11	UNKNOWN	4,65	0,16	0,3	0,0	0,159	7,71
12	UNKNOWN	6,19	0,22	0,4	0,0	0,219	10,59
13	UNKNOWN	6,72	0,31	0,2	0,0	0,309	11,58
14	UNKNOWN	6,97	0,10	0,1	0,0	0,103	12,06
Total			100,00	65,7	7,2	100,000	69,87

Voclosporine

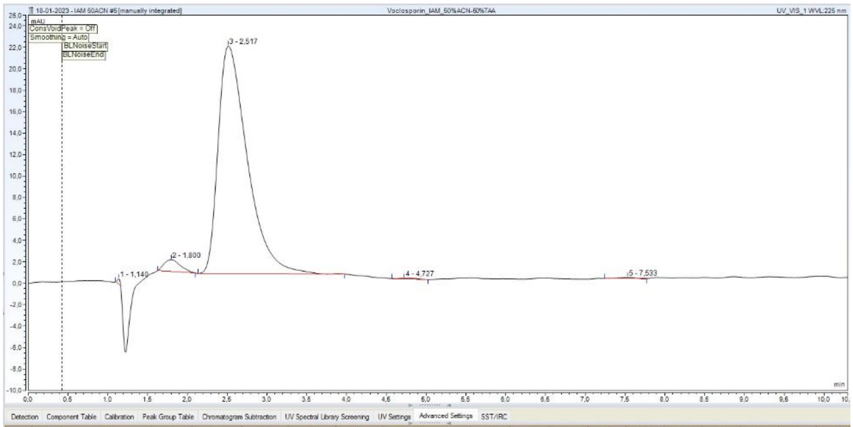

	A	B	C	D	E	F	G	H	I	J	K	L
1	Peak	Peak Name	Ret.Time	Amount	Rel.Area	Area	Height	Type	Width (50%)	Asym.	Resol.	Plates
2	No.		min	n.a.	%	mAU*min	mAU		min	EP	EP	EP
4	1		1,140	n.a.	0,19	0,0178	0,49	BMB*	0,037	0,83	2,91	5352
5	2		1,800	n.a.	2,80	0,2615	1,10	BMB*	0,231	1,32	1,38	336
6	3		2,517	n.a.	96,63	9,0159	21,25	BMB*	0,382	1,72	4,15	241
7	4		4,727	n.a.	0,15	0,0135	0,06	BMB*	0,247	1,78	7,13	2028
8	5		7,533	n.a.	0,23	0,0216	0,10	BMB*	0,218	0,85	n.a.	6641
9	Maximum			0,0000	96,63	9,0159	21,25		0,382	1,78	7,13	6641
10	Minimum			0,0000	0,15	0,0135	0,06		0,037	0,83	1,38	241
11	Sum			0,0000	100,00	9,3303	22,99					

Figure S5. *HPLC purity assessment for the studied compounds following the order of table S6. Voclosporin is placed at the end.*
